# Loss of *Neogenin1* in human colorectal carcinoma cells causes a partial EMT and wound-healing response

**DOI:** 10.1038/s41598-019-40886-y

**Published:** 2019-03-11

**Authors:** Vishal Chaturvedi, Alexandre Fournier-Level, Helen M. Cooper, Michael J. Murray

**Affiliations:** 1 School of Biosciences, The University of Melbourne, Melbourne, Victoria 3010 Australia; 2 The University of Queensland, Queensland Brain Institute, Brisbane, Queensland, 4072 Australia

## Abstract

Neogenin1 (NEO1) is a receptor of the Deleted in Colorectal Carcinoma (DCC)/Frazzled/UNC-40 family, which regulates axon guidance but can also stabilize epithelial adherens junctions. NEO1 and DCC are also tumor suppressors that can inhibit metastasis by acting as dependence receptors. Given the role of NEO1 in maintaining adherens junctions we tested whether loss of NEO1 also promoted metastasis via an epithelial mesenchymal transition (EMT). Loss of NEO1 disrupted zonula adherens but tight junctions were unaffected. *Neo1-*depleted epithelial cells exhibited a more migratory morphology, had reduced F-actin rich stress-fibres and more basal lamellipodia. Microtubule density was decreased while microtubule outgrowth was faster. Live imaging showed that *Neo1*-depleted epithelial islands had increased lateral movement. Western blots and immunostaining revealed increased expression of mesenchymal markers such as Fibronectin and MMP1. Furthermore, RNA-seq analysis showed a striking decrease in expression of genes associated with oxidative phosphorylation, and increased expression of genes associated with EMT, locomotion, and wound-healing. In summary, loss of NEO1 in intestinal epithelial cells produces a partial EMT response, based on gene expression, cellular morphology and behaviour and cytoskeletal distribution. These results suggest that loss of NEO1 in carcinomas may contribute to metastasis by promoting a partial EMT and increased motility.

## Introduction

Receptors of the Neogenin/Deleted in Colorectal Carcinoma (DCC)/Frazzled/UNC-40 family were originally identified as axon guidance receptors^1^ but have been increasingly linked to epithelial morphogenesis events^2^. For example, in the terminal-end buds of the developing mammary gland, NEO1 is required for adhesion between cap cells and luminal cells^3^. NEO1 also promotes the apico-basal polarisation of neuroepithelial cells during neural tube formation in Xenopus^4^. Neo-family receptors also regulate epithelial events in *Drosophila*. Frazzled, the Neo/DCC ortholog, inhibits a partial epithelial-mesenchymal transition (EMT) event that occurs during pupal metamorphosis^5^ and also promotes the formation of the gut epithelium in the embryo^6^. More recently, two studies have demonstrated a role for NEO1 in maintenance of adherens junctions (AJs). In the Caco-2 colorectal cancer cell line, NEO1 localises to AJs in an E-Cadherin (E-Cad) dependent way, where it promotes F-Actin formation and junctional tension by recruiting the WAVE Regulatory Complex (WRC) and Arp2/3^7^. Similarly, *in vivo*, NEO1 acts through the WRC to maintain junctions in the radial progenitors of the embryonic cortex and the ependymal epithelium lining the ventricles in the brain^8^.

As might be expected for genes that promote epithelial stability, loss of Neo-family receptors is strongly linked to metastasis. For example, low levels of NEO1 expression correlate with malignancy of breast^9^ and lung cancers^10^, and promote migration and invasion of glioblastoma cells^11^, while loss of DCC expression is common in colorectal cancers^12^. Exactly how loss of DCC and NEO1 contribute to metastasis is unclear. While loss of epithelial junctions and polarity could play a causative role, inhibition of apoptosis is also strongly implicated. Both, DCC and NEO1 can act as dependence receptors that induce apoptosis when deprived of their ligands^13,14^. Thus, a cancer cell that has lost its NEO1/DCC receptor is thought to be able to move away from its normal environment with impunity^15^.

Here, to gain insight into how loss of NEO1 affects epithelial cells, we have examined the transcriptional changes and the cellular phenotypes associated with *Neo1* knockdown in Caco-2 cells. To examine the role of *Neo1* during formation of an epithelium, siRNA transfection was carried out prior to cell seeding. Loss of NEO1 resulted in a cell-cell junction “blebbing” phenotype whereby the tight apposition of cells at the zonula adherens was disrupted, and basal F-Actin rich stress fibres were lost as previously described^7^. We now show that *Neo1* depleted cells also have sparsely populated microtubules (MTs) and longer and faster EB1 comets. RNA-seq analysis of *Neo1* knockdown cells revealed a striking shift in transcriptional profile consistent with a partial EMT. In addition, however, many upregulated genes are consistent with a response to damage of the intestinal epithelium. Upregulated gene sets include those involved in locomotion, wound healing, response to luminal microbial pathogens, stress-response and extracellular matrix (ECM) remodelling. Many of the upregulated genes are also strongly implicated in promoting metastasis again consistent with a partial EMT signature. Interestingly, genes that were down-regulated are strongly enriched for those involved in oxidative phosphorylation. These results confirm the importance of NEO1 in maintaining epithelial integrity and provide insight into the transcriptional response of intestinal epithelial cells when cadherin-dependent adhesion is disrupted.

## Results

### Neo1 knockdown disrupts the zonula adherens and stress-fibres

**Figure 1:**
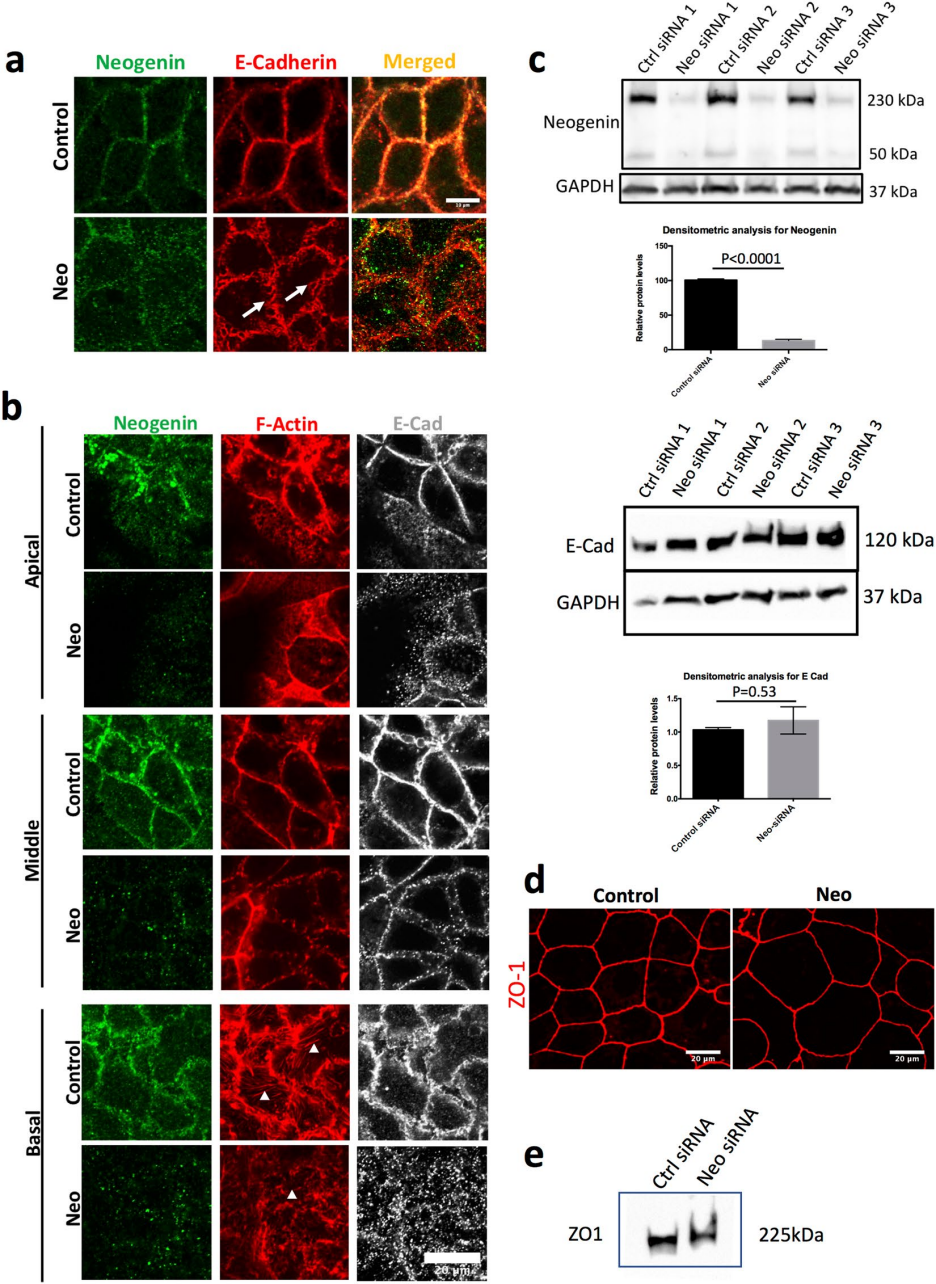
*Neo1* knockdown disrupts adherens junctions and cytoskeletal integrity in Caco-2 cells. (**a**) Caco-2 cells treated with control or *Neo1*-siRNA and immunostained for NEO1 (green) and E-Cad (red) showed disruption of the zonula adherens (arrows). The image is a maximum projection image of a z-stack (1 μm). Scale bar-20 μm (**b**) Caco-2 cells stained for NEO1 (green), F-actin (red) and E-Cad (grey). Images at three apico-basal positions (1 μm, 4 μm and 8 μm) showing disruption of E-Cad localisation and reduction of basal F-Actin rich stress fibres (arrowheads). Scale bar-20 μm (**c**) *Neo1* knockdown in Caco-2 cells was confirmed by Western blot and densitometric analysis. Representative blot with three biological replicates from one experiment and Neogenin blot has been stripped and reprobed for GAPDH. Full length blots for Neogenin and GAPDH are shown in Supplementary Fig. S1. No significant change in E-Cad protein levels after *Neo1* knockdown. Each band represents cell lysate proteins from a biological replicate from three independent experiments and E-Cad blot has been stripped and reprobed for GAPDH. Full length blots for E-Cad and GAPDH are shown in Supplementary Fig. S2. (**d**) Tight junctions were not disrupted after *Neo1* knockdown as can be seen with continuous ZO-1 staining (red). Scale bar-20 μm. (**e**) Western blot for ZO-1 in control and *Neo1*-siRNA treated cells. Full length blot for ZO-1 is shown in Supplementary Fig. S3.

The efficacy of *Neo1-*siRNA and its effect on AJs was confirmed by repeating the method of Lee *et al*.^7^. *Neo1-*siRNA was transfected into 1-day old epithelia (hereafter referred to as post-transfection) and cells were immunostained after 2 days for E-Cad and NEO1. *Neo1* knockdown reduced NEO1 protein levels by ~90% (Fig. 1c and Supplementary Fig, S1) and, as before^7^, disrupted AJs, causing membrane blebs to appear (Fig. 1a, arrows). However, we did not see any significant change in the levels of total cellular E-Cad protein (Fig. 1c and Supplementary Fig. S2). To investigate the effects of earlier knockdown of *Neo1*, Caco-2 cells were co-transfected with *Neo1*-siRNA before being seeded (hereafter referred to as co-transfection), cultured for 5 days and then stained for E-Cad, F-Actin and NEO1. As previously reported F-actin showed thick actin bands at the AJs in the sub-apical and middle regions of the cells while, basally, the cells were rich in stress fibres (Fig. 1b, arrowheads). In *Neo1*-siRNA treated cells actin localisation was largely intact in the most apical regions but became weaker in middle regions, and there was a clear reduction in stress fibres at the basal side of cells (Fig. 1b, arrowheads).

E-Cad in co-transfected cells showed similar though more severe effects than post-transfected cells. In control-siRNA treated cells E-Cad showed a linear arrangement at the zonula adherens (ZA) along cell-cell contacts, and little cytoplasmic E-Cad. In contrast, *Neo1*-siRNA treated cells showed bleb-like structures at the interface of cell-cell junctions with the blebs projecting into the cytoplasm (Fig. 1b) and E-Cad was largely lost from cell-cell junctions and was instead localised to extensive puncta (Fig. 1b). Localisation of the tight junction marker ZO-1 appeared unaffected (Fig. 1d) and protein levels were also unaffected (Fig. 1e and Supplementary Fig. S3) as previously found^7^.

We also tested the effects of *Neo1* knockdown on three other CRC cell types: SW480, DLD-1 and RKO. qPCR results showed that each of these lines expressed *Neo1* at levels similar to, or higher than, Caco2 cells (Supplementary Fig. S4) but with no appreciable expression of DCC as expected. These cell lines, when grown to confluency showed a wide variation in phenotype and the degree of epithelial-mesenchymal characteristics (Supplementary Fig. S4). DLD-1 cells were most clearly epithelial with clear ZAs in apical regions, having both F-Actin and E-Cad, and F-Actin stress-fibres in basal regions. However, junctional E-Cad was much weaker than in Caco-2 cells, and much of the E-Cad was localised to cytoplasmic puncta. SW480s were more mesenchymal with only F-Actin at the cell-cell junctions while E-Cad was confined to puncta. RKOs were most mesenchymal with no obvious cell-cell junctions. Both SW480 and RKO cells showed extensive basal ruffles and no stress-fibres. *Neo1* knockdown had no obvious effects on any of these phenotypes suggesting that only in epithelia with strong junctional tension, such as Caco-2 cells^7^, does Neo have a key role.

These results confirm that loss of *Neo1* specifically disrupts the ZA in Caco-2 cells.

### Neo1-depleted cells exhibit a distinct genomic expression profile

Next, to investigate the effects of *Neo1* knockdown on gene expression, we performed a whole-transcriptome analysis of both co-transfected and post-transfected Caco-2 cells. Cells were either co-transfected with control or *Neo1* siRNA, propagated for 5 days, and RNA extracted, or cells were post-transfected with control or *Neo1*-siRNA 1 day after seeding and RNA collected after 2 days. Gene-expression changes associated with *Neo1*-knockdown in post-transfected cells were similar to co-transfected cells, but less dramatic (Supplementary Table S1), so the results described below refer to co-transfection cells unless otherwise stated.

**Figure 2:**
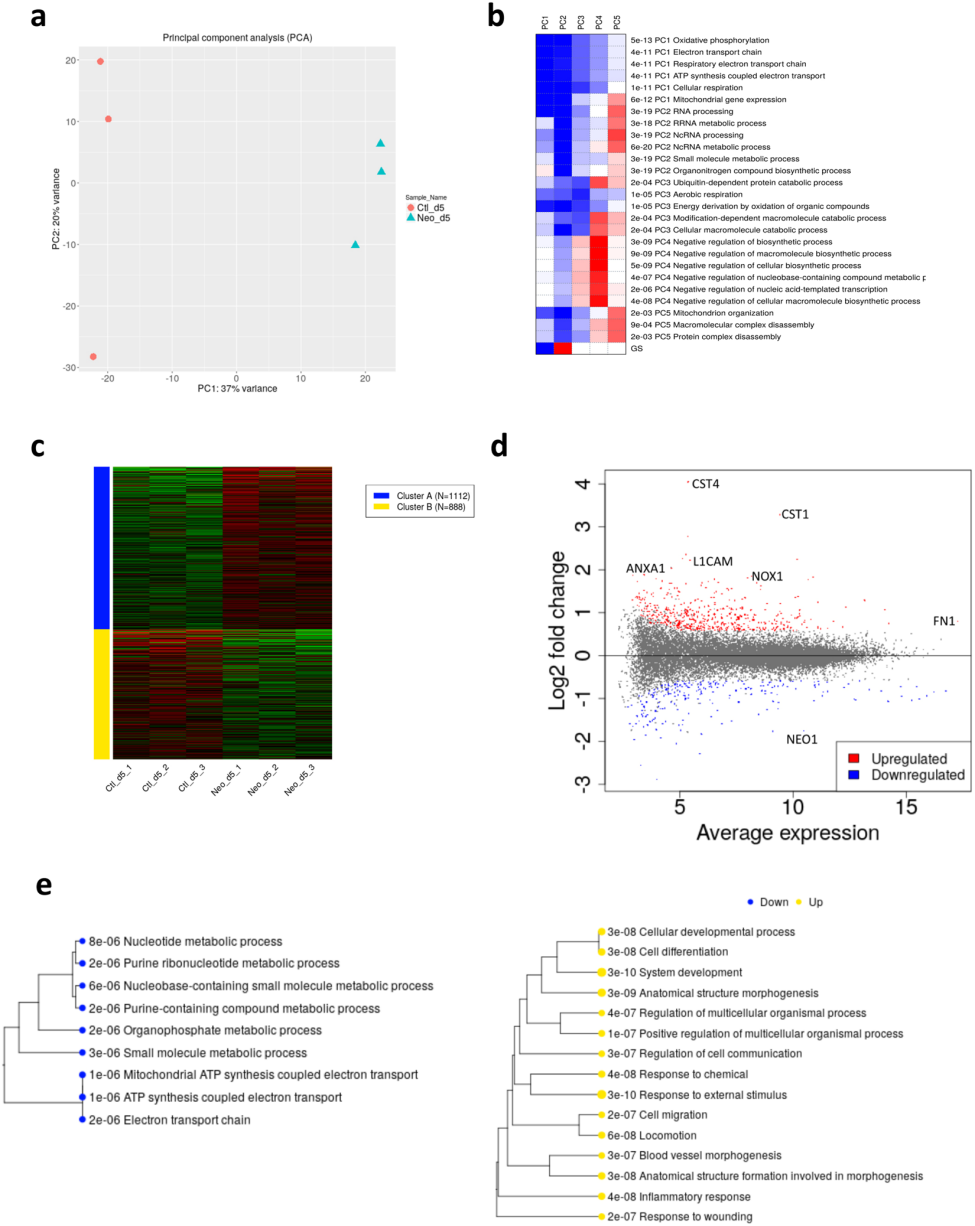
Whole transcriptome analysis of Caco-2 cells after *Neo1* knockdown. **(a**) Principal component analysis (PCA) plot of Control and *Neo1*-siRNA treated cells showed 37% of variance in the first axis. (**b**) GO-term enrichment analysis of PCA loadings for each gene show the majority of the variance is associated with down-regulation of the oxidative phosphorylation pathway. (**c**) K-means clustering of the top 2000 variable genes clearly resolved in two clusters of genes up or down-regulated. (**d**) MA-plot of the 419 up-regulated and 170 down-regulated genes, significantly altered by *Neo1* knockdown. (**e**) GO-term enrichment analysis of differentially expressed genes categorized into down-regulated and up-regulated pathways.

Gene expression data was uploaded to the iDEP web interface (integrated Differential Expression and Pathway Analysis) and 18,282 genes included for further analysis (see Materials and Methods for details). PCA analysis confirmed that there was extensive variation among control and *Neo1*-siRNA (separated along PC1 accounting for 35 to 37% of variance in expression; Fig. 2a) and less so among replicates (separated along PC2 accounting for 18 to 20% of the variance).

Pathway analysis based on the loadings along PC1 showed that the primary separation between *Neo1* siRNA treated cells and control cells was due to downregulation of genes involved in oxidative phosphorylation (Fig. 2b). K-means clustering analysis, using the most variable 2000 genes, distinguished two clusters of expression profile between *Neo1* siRNA and control cells (Fig. 2c). Gene Ontology (GO) term analysis also showed that the cluster of genes with decreased expression in *Neo1-*depleted cells was enriched for oxidative phosphorylation activities while the cluster of genes with increased expression was enriched for wound healing response activities and other pathways (see below). Increasing the number of clusters beyond two did not produce any further sub-clusters with significant pathway enrichment.

### Neo1 knockdown results in downregulation of cellular respiration genes

To further investigate enriched pathways, differential gene expression analysis was performed with iDEP. This identified a total of 419 upregulated and 170 downregulated genes when *Neo1* was knocked down. Figure 2d shows the MA plot of genes with significantly altered expression. Enrichment analysis for GO term associated with Biological Processes was conducted for this list of genes using an adjusted p-value <1e-06 (Fig. 2e).

Among the down-regulated genes, small-molecule metabolism pathway genes were overrepresented (Supplementary Table S2). This included 10 mitochondrial genes of the electron transport chain (*MT-CO1*, *MT-CO2*, *MT-CO3*, *MT-CYB*, *MT-ND1*, *MT-ND2*, *MT-ND4*, *MT-ND4L*, *MT-ND5*, *MT-ND6*), these genes were already identified as highly variable in the k-means analysis. Association with mitochondria was also seen in the GO term enrichment for Cellular Components with 29 genes associated with mitochondrion (adj. p-value < 1.8e-06), 20 of which associated with the inner mitochondrial membrane (p-value = 2.7e-09). Oxidative phosphorylation activity was also enriched among downregulated genes in the post-transfected *Neo1*-siRNA cells (Supplementary Table S1).

**Figure 3:**
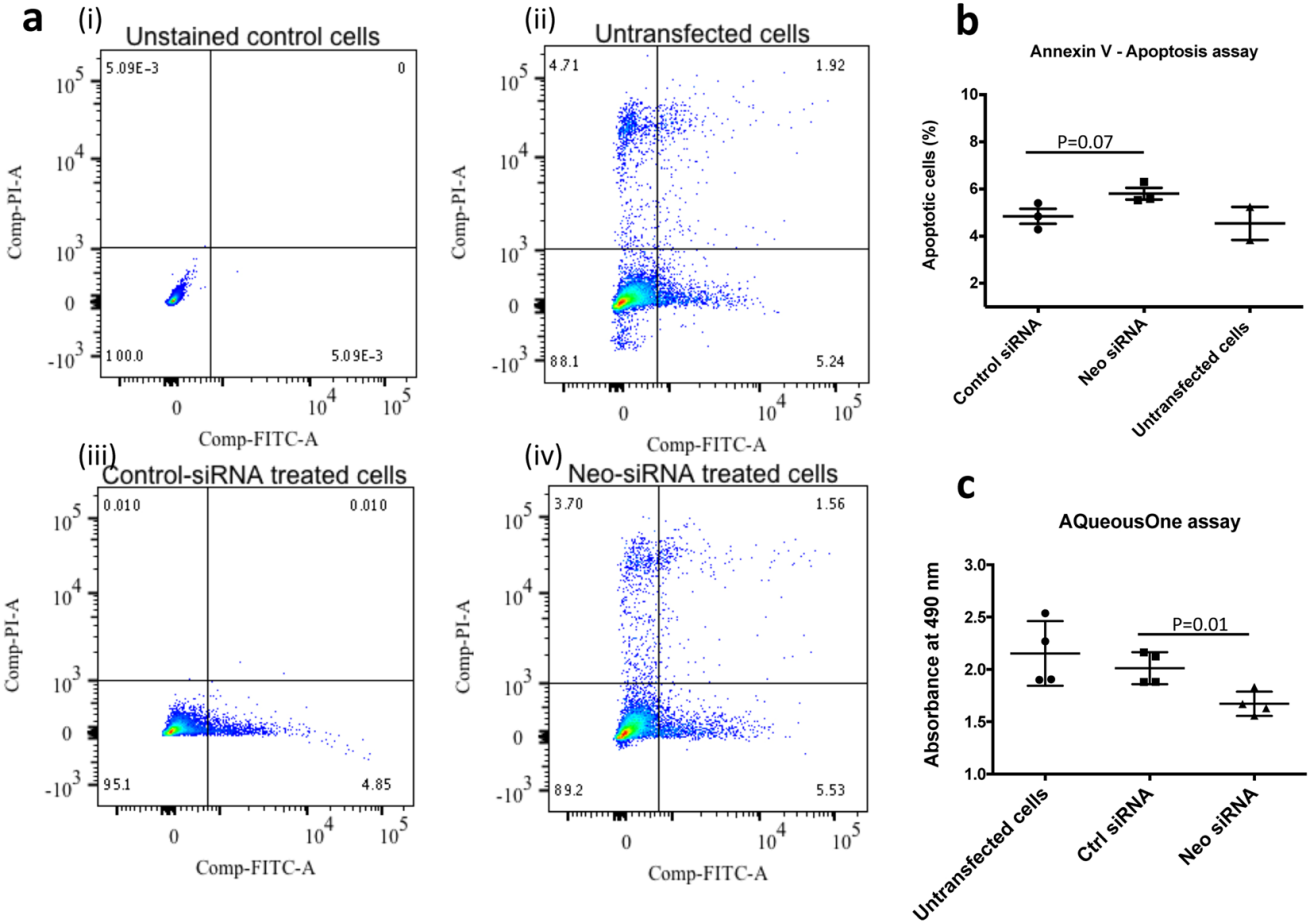
Apoptosis and metabolic assay. (**a**) Annexin-V FITC apoptosis assay on control and *Neo1*-siRNA treated cells showed no significant difference in apoptotic cells after *Neo1* knockdown (i) Unstained cells (ii) Control cells with no siRNA treatment (iii) Control siRNA treated cells (iv) *Neo1* siRNA treated cells (**b**)% of apoptotic cells p-value = 0.07. (**c**) AQueous One metabolic assay showed a significant decrease in the metabolic activity of cells after *Neo1* knockdown. p-value = 0.01.

Since NEO1 can act as a dependence receptor^16^ and apoptosis can cause mitochondrial damage^17^, we checked whether the reduced expression of mitochondrial genes might be associated with increased apoptosis. Levels of apoptosis, as determined by an Annexin V apoptosis assay, showed no significant difference between *Neo1* (5.80 ± 0.24) and control-siRNA (4.84 ± 0.32) treated cells (p-value = 0.073 Fig. 3a,b). However, the level of cell metabolism, tested via the AQueous One proliferation assay showed a significant decrease in absorbance from control-siRNA treated to *Neo1*-siRNA treated cells (2.01 ± 0.07 vs 1.67 ± 0.05, respectively; p-value = 0.01) suggesting a reduction in metabolic activity of *Neo1* knockdown cells and hence a reduced rate of proliferation (Fig. 3c). We did not see a significant change (p-value = 0.44) in metabolic activity of control cells (untransfected) compared to control siRNA treated cells (2.15 ± 0.15) confirming that transfection did not reduce cell metabolism.

### Neo1 knockdown results in a general wound-healing response

Pathways enriched for significantly upregulated genes in *Neo1* knockdown cells represented several broad categories including development/differentiation/morphogenesis (149 genes), cell migration/locomotion (61 genes), response to stress/chemicals/external stimuli (160 genes) and wound-healing (36 genes) (Supplementary Table S2). Enrichment in K-means clusters produced similar results with upregulation of pathways associated with response to wounding (42 genes), angiogenesis and blood vessel development (37 genes), inflammatory response (45 genes) and extracellular matrix (ECM) organisation (28 genes) (Supplementary Table S3). A subset of these pathways was over represented among genes upregulated in the post-transfected cells except the wound healing set (Supplementary Table S1).

Also, enrichment analysis within the “Hallmarks” gene sets from the Molecular Signatures Database v6.1^18^ returned as its top hit “Epithelial Mesenchymal Transition” (adj. p-value = 9.0e-10) with 20 genes included (Supplementary Table S2). These genes included several associated with the ECM and cytoskeleton, as well as TGFB1, a ligand of the well known TGF beta EMT pathway. Interestingly several other TGF-beta superfamily genes were significantly upregulated (TGFB3, INHBE, INHBB, NRTN) as was the receptor TGFBR2. KEGG pathway analysis identified the top three pathways as the PI3K-Akt signaling pathway, the MAPK signaling pathway and ECM-receptor interactions, all of which are linked to EMT (Supplementary Table S2).

In addition to the EMT-signature, however, many of the upregulated genes could also be interpreted as a response to wounding and defence against extracellular pathogens. For example, two of the most significantly upregulated genes were the cystatins (*CST1* and *CST4*), which are type 2 secreted cysteine-proteinase inhibitors found in fluids holding anti-bacterial and anti-viral properties such as saliva^19^. Other upregulated genes included lysozyme (*LYZ*), an anti-bacterial agent found in saliva, NPPB, a secreted natriuretic peptide with anti-bacterial and anti-fungal activity^20^, SLP1, a secreted serine-protease inhibitor that protects epithelial surfaces and has anti-bacterial, anti-viral and anti-fungal properties^21^, TFF1, a stable trefoil-family protein found in the gastrointestinal mucosa that is thought to protect the epithelium and aid in healing^22^ and A2M, a secreted protease inhibitor that can inhibit trypsin, thrombin and collagenase^23^. There were also 9 genes associated with the interferon response (*IFI6*, *IFITM1/3*, *IFRD1*, *ISG15*, *OAS1*, *SOCS2*, *TGFB1* and *TMEM2*) and several genes associated with platelet degranulation and coagulation (Supplementary Table S2).

Consistent with both the partial EMT signature, and a defence response to external factors, analysis of Cellular Component GO terms showed highest enrichment for genes associated with the Extracellular space (112 genes, p-value = 1.4e-10, Supplementary Table S2).

We speculate that loss of ZA-integrity triggers a general intestinal wound response involving both epithelial closure processes, secretion of anti-microbials and inflammation.

### Neo1 knockdown results in increased expression of genes regulating cell locomotion and ECM remodelling

Many of the most significantly upregulated genes in *Neo1* knockdown cells fell into pathways associated with cell locomotion and ECM remodelling. The strongest upregulation was a 2.3 log2-fold increase in L1 cell adhesion molecule (*L1CAM*), a transmembrane glycoprotein of the Ig superfamily that has been shown to promote tumour cell invasion and motility^24^.

We also found a 1.8 log2-fold increase in both *ANXA1* and *NOX1* levels in *Neo1* knockdown cells. These two genes collaborate in an epithelial repair pathway known to operate in the intestinal mucosa^25^. ANXA1 is a ligand for N-formyl peptide receptors (FPRs) whose activation leads to increased production of reactive oxygen species (ROS) by the epithelial NADPH oxidase NOX1, which in turn promotes epithelial movement and repair^25^. Similarly in a study conducted by Kato *et al*.^26^ using a mouse model of experimental colitis, *Nox1* deficient mice showed a reduced recovery of mucosal epithelium following dextran sulphate-induced colitis which was due to inhibition of proliferation, migration and survival of crypt progenitor cells^26^. *Nox1* overexpression has also been related to the progression of human colon cancers^27^.

Fibronectin (*FN1*) and integrins (alpha-2, alpha-5 and beta-1) were also upregulated. *FN1* is a key component of the ECM while integrins are key cell surface receptors for fibronectins, collagens, laminins and other molecules of the ECM. Integrins play a major role in cell adhesion, actin cytoskeleton reorganization and migration. Moreover, upregulation of Integrins alpha-5 beta-1 have been associated with enhanced cell migration and malignant phenotypes in ras-transformed mammary epithelial cells (EpH4)^28^. Similarly, *L1CAM* can promote tumour cell invasion and motility, possibly by increased *L1CAM-beta1* integrin interactions and *L1CAM-beta1* internalization and recycling^29^.

**Figure 4:**
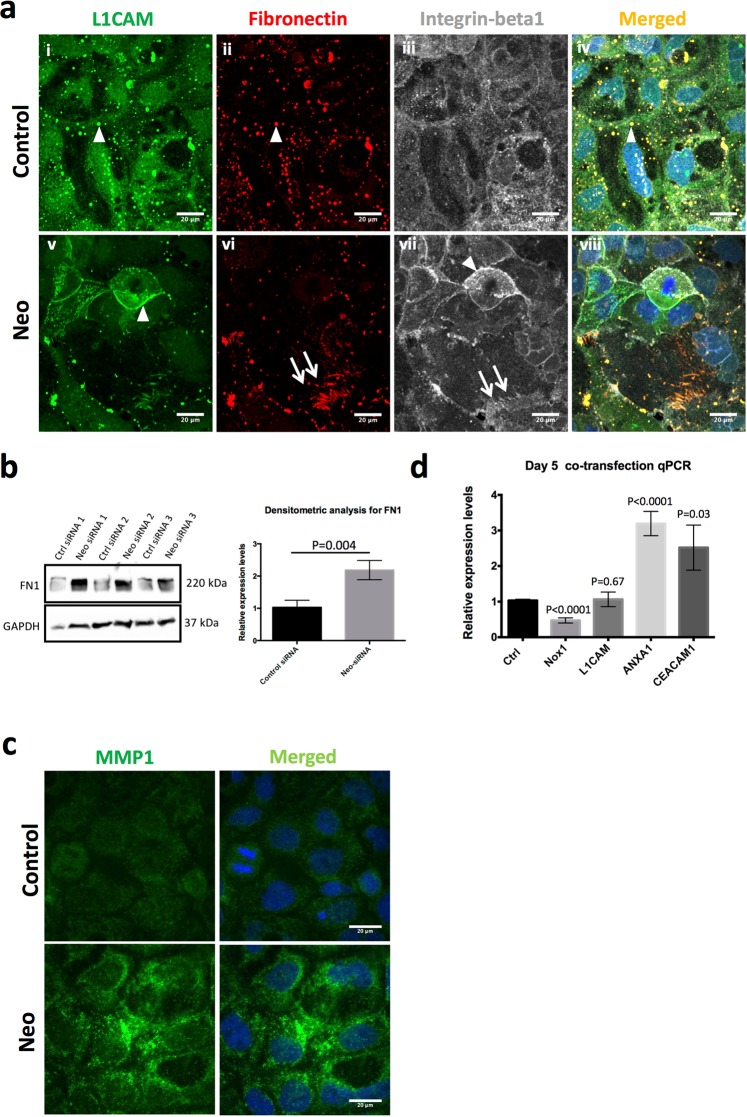
Validation of RNA-seq analysis. (**a**) Immunofluorescence staining of Caco-2 cells showed enhanced L1CAM (green, arrow in v) and integrin beta-1 expression (gray, arrow in vii) in *Neo1* transfected cells (v–viii) when compared to control cells (i–iv). Fibronectin (red, arrowhead in ii) was more globular in control cells while fibrils are clearly visible in *Neo1* transfected cells (red, arrow in vi) suggesting cell migration. Co-localization (yellow) of L1CAM (green, arrowhead in i) and fibronectin (red, arrowhead in ii) can also be clearly seen in endosome-like structures. Scale bar-20 μm. (**b**) Western blots and densitometric analysis show increased FN1 expression in Caco-2 cells after *Neo1* knockdown. Each band represents cell lysate proteins from a biological replicate from three independent experiments. Full length blot for FN1 and GAPDH are represented in Supplementary Fig. S5. (**c**) Immunofluorescence staining of Caco-2 cells reveals the vesicular distribution of intracellular MMP1 and also showed enhanced staining in *Neo1* treated cells, Scale bar-20 μm. (**d**) q-PCR validation of RNA-seq analysis. RT-qPCR of top 4 genes under cell migration category after *Neo1* knockdown in co-transfected Caco-2 cells shows significant upregulation of *CEACAM1*, *ANXA1* and downregulation of *NOX1* relative to the reference gene (*TBP*). Data represent mean ± SEM of 6 biological replicates. p-value = 0.03 for *CEACAM1*, p-value < 0.0001 for *ANXA1* and *NOX1*. *L1CAM* levels were not significantly changed, p-value = 0.67 (two-tailed student’s t-test).

To see if these transcriptional changes were accompanied by changes in protein levels and localisation we examined FN1 (Red), the integrin ITGB1 (Gray), and L1CAM (Green) expression in control and *Neo1-*depleted cells at 3-days post-transfection. FN1 deposition in control cells was more globular while *Neo1*-siRNA transfected cells showed fibrils deposited at the basal surface which coincided with integrin beta-1 positive filopodia like structures (arrows in Fig. 4a,vi and vii) implying migratory behaviour of the cells. We also observed clear co-localization of FN1 and L1CAM in endosome-like structures (arrowheads in Fig. 4a,i, ii and iv). FN1 localisation to endosome like structures and fibrils has also been reported by other researchers^30^. ITGB1 was distributed ubiquitously through the cells. Interestingly, while L1CAM localisation was only seen in cytoplasmic globules in control cells, *Neo1*-siRNA treated cells showed enhanced L1CAM staining at the cell-surface in addition to the globular staining. Furthermore, cells with enhanced L1CAM staining also had regions of increased ITGB1 staining which, interestingly, tended to be complementary to those regions enriched for L1CAM (arrowheads in Fig. 4a,v and vii). Also, these cells appeared to be sitting slightly above than other cells within the epithelial island suggesting that increased expression of these cell adhesion molecules was associated with increased motile activity (data not shown).

Western blots for FN1 confirmed our immunostaining results where *Neo1*-siRNA cells showed a significant 2.24-fold upregulation of FN1 total cellular protein levels (p-value = 0.004) (Fig. 4b and Supplementary Fig. S5). Matrix metalloproteinases (MMPs) are enzymes that digest the ECM at the invading front of the cells and MMP1 has been shown to promote the invasion and tumorigenesis of breast cancer cells^31^, hence we also investigated the intracellular MMP1 levels of control and *Neo1*-siRNA treated cells by immunostaining (Fig. 4c). Our results showed vesicular staining for intracellular MMP1 and the levels were increased in Neo1-siRNA treated cells further suggesting cell migration.

To further validate our RNA-seq results we performed qPCR on the top 4 genes in the Locomotion pathway (Fig. 4d). These genes were – *L1CAM*, *CEACAM1*, *ANXA1* and *NOX1*. We found *CEACAM1* and *ANXA1* were significantly upregulated (2.51x, p-value = 0.03 and 3.19x, p-value < 0.0001 respectively) in *Neo1*-siRNA treated cells while there was only a slight increase in the *L1CAM* levels which was not significant (1.06x, p-value = 0.77). Surprisingly, there was a significant reduction in *NOX1* levels (0.47x, p-value < 0.0001).

### Neo1 knockdown epithelial islands exhibit a mesenchymal phenotype

**Figure 5:**
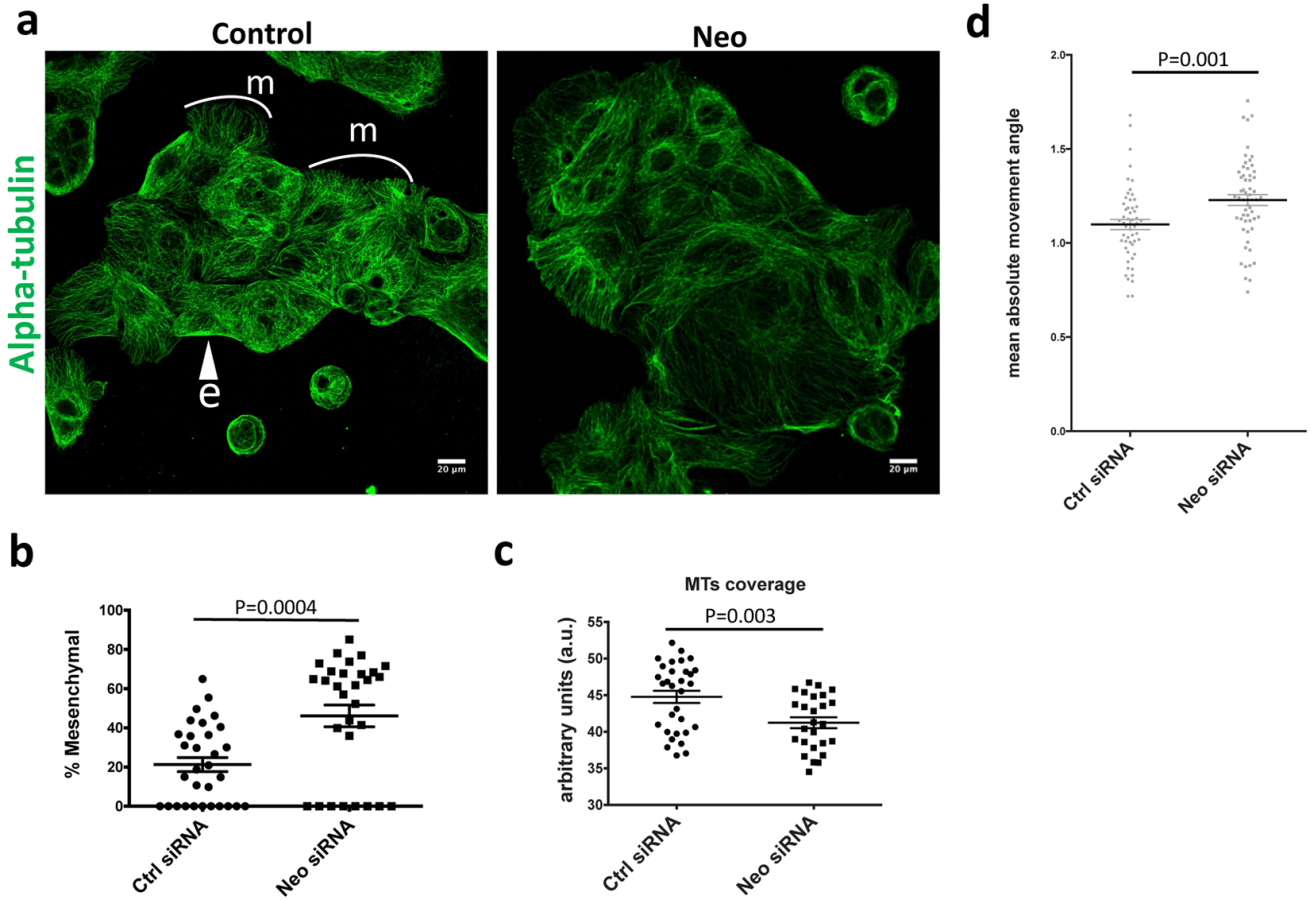
Phenotypic characterization of *Neo1*-depleted Caco-2 epithelial islands. (**a**) Immunostaining of control and *Neo1*-siRNA treated Caco-2 epithelial islands with alpha-tubulin (green). A proportion of cells around the periphery of islands exhibited a spread, migratory morphology in which microtubule arrays were oriented away from the island. These were categorized as “mesenchymal” (m). Loss of *Neo1* resulted in a higher proportion of mesenchymal cells and decreased microtubule density. (**b**) Quantification of the proportion of mesenchymal cells per island, p-value = 0.0004. (**c**) Quantification of mean MT density in control and *Neo1-*depleted cells (see Materials and Methods for details), p-value = 0.003. (**d**) Kymograph analysis of *Neo1*-knockdown Caco-2 cells revealed an increase in the angle of movement of internal cellular features, p-value = 0.001.

Given the partial EMT signature seen in RNA-seq data and increased deposition of FN1 and MMP1 in our cellular analysis, we further investigated the cellular morphology and cytoskeleton of *Neo1-*siRNA cells as they were forming an epithelium. Caco-2 cells were co-transfected with Control/*Neo1*-siRNA and 24 hours later were fixed and stained for DAPI and alpha-tubulin. In this assay we found that the cells at the edges of epithelial islands exhibited two distinct morphologies and MT distributions. Some cells, which we will refer to as “epithelial”, had dense concentric MTs and a smooth cellular edge profile. Others, which we will call “mesenchymal”, had a more migratory/lamellipodial morphology and long MTs projecting outwards (Fig. 5a). We scored the proportion of “mesenchymal” vs “epithelial” cells in islands of at least 5 cells. The majority of islands were in the range of 10–25 cells.

Control-siRNA islands generally tended to be more epithelial with an average of 21.26% ± 3.603 (N = 31 islands) peripheral cells showing a mesenchymal phenotype (Fig. 5b). Conversely, for *Neo1*-siRNA islands, 46.08% ± 5.544 (N = 30 islands) of peripheral cells were mesenchymal (p-value = 0.0004). Similar results were obtained when the fraction of the perimeter length of mesenchymal versus epithelial type was measured (Supplementary Fig. S6, p-value < 0.0001).

In addition to the increased proportion of cells exhibiting a mesenchymal phenotype we observed a decrease in the density of the microtubules in *Neo1* siRNA treated. Using an automated ridge-detection algorithm (see Materials and Methods for details) we found the MT density decreased from 44.76 ± 0.8393 a.u. (N = 31 islands) (arbitrary units) in Control-siRNA cells down to 41.23 ± 0.7383 a.u. (N = 26 islands) in *Neo1-*siRNA cells (Fig. 5c).

To see if *Neo1-*siRNA islands exhibited differences in cellular movement, we next collected time-lapse images at 15-sec intervals for a period of 30 min. In this short time period in *Neo1*-siRNA treated cells we could detect a “sliding” movement of the cells within the island and at the edge (Supplementary Movie S2) - suggesting a shift towards mesenchymal-migratory phenotype while control cells appear more stable (Supplementary Movie S3). Quantification of these movements using a kymograph analysis (see Materials and Methods for details) showed a significant increase in cell movement suggesting that connections with the underlying ECM were more labile (Fig. 5d).

Overall the results show that loss of *Neo1* results in a partial EMT response and increased epithelial migration.

### *Neo1*knockdown led to an increase in the EB1 comet length

Given the loss of ZA integrity, increased mesenchymal behaviour and reduced microtubule density we next examined microtubule dynamics using the plus-end marker EB1.

**Figure 6:**
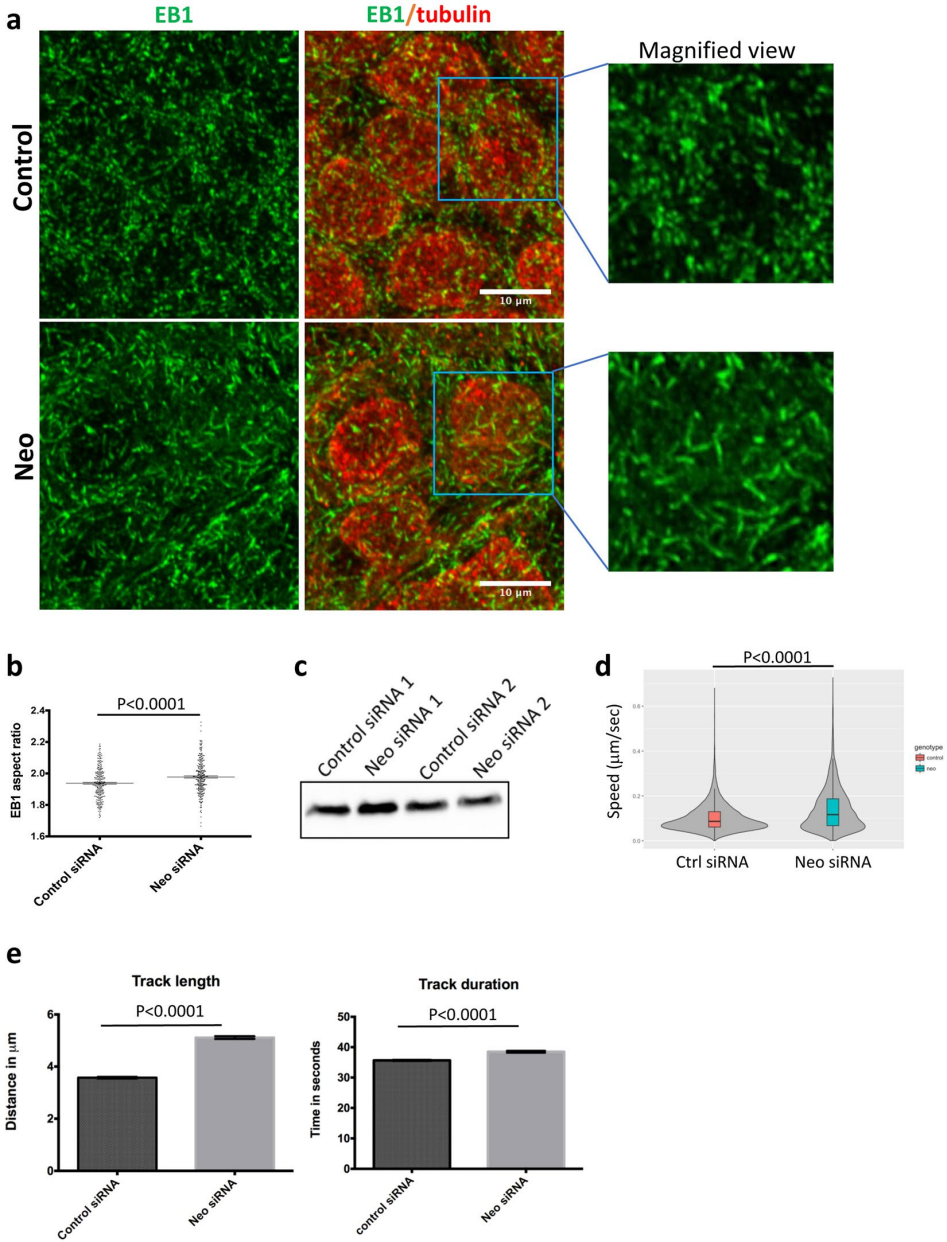
*Neo1* knockdown results in longer and faster comets of the microtubule plus end binding protein EB1. (**a**) Immunostaining of Caco-2 cells for EB1 (green) and alpha-tub (red). (**b**) Morphometric analysis of EB1 comets showed significantly higher aspect ratio (i.e. longer EB1 comets in *Neo1*-knockdown cells (1.97) compared to controls (1.93), p-value < 0.0001. (**c**) Western blots of EB1 shows no significant change in total protein levels in control and *Neo1*-siRNA treated cells. Full length blots for EB1 are represented in Supplementary Fig. S7. (**d**) EB1 comets speed analysis using Imaris showed faster EB1 comets (0.13 μm/sec) after *Neo1* knockdown as compared to control cells (0.10 μm/sec), p-value = 0.0001. (**e**) EB1 comets track length and duration was significantly longer in *Neo1*-siRNA treated cells, p-value < 0.0001.

Caco-2 cells were seeded in 35 mm iBiDi culture dishes and 24 h later were transfected with *Neo1*-siRNA. After 48 h of transfection, cells were fixed, permeabilized and immunostained with anti-EB1 and anti-tubulin antibodies (Fig. 6a). EB1 staining in control and *Neo1* siRNA treated cells revealed a comet like pattern distributed throughout the cytoplasm of the cell and also concentrically around the nuclei. However, EB1 comets in *Neo1-*depleted cells appeared more elongated than in controls (Fig. 6a). To quantify this, a mean aspect ratio of comets was determined for each cell (see Materials and Methods) and averaged over multiple cells. Comets in *Neo1-*depleted cells had a significantly greater aspect ratio than those in control siRNA treated cells (1.97 ± 0.006, N = 273 cells; versus 1.93 ± 0.006, N = 299 cells; p-value < 0.0001) (Fig. 6b). The protein levels of EB1 were similar in control and *Neo1* siRNA treated cells (Fig. 6c and Supplementary Fig. S7).

Finally, since longer EB1 comets might correlate with increased microtubule dynamics we transfected cells with a plasmid encoding EB1-GFP and captured time-lapse images of EB1-GFP comets at every 2 sec. As expected the speed of microtubule growth was significantly greater in *Neo1-*siRNA treated cells, increasing from 0.10 μm/sec (Control, Supplementary Movie S5) to 0.13 μm/sec (*Neo1*, p-value < 0.0001, Supplementary Movie S4) (Fig. 6d). In addition, mean duration and displacement of comets was also significantly greater in *Neo1* siRNA (p-value < 0.0001, Fig. 6e).

Thus, loss of *Neo1* creates a significant shift in the microtubule cytoskeleton with fewer microtubules and increased microtubule dynamics.

## Discussion

Our results show that loss of *Neo1* in the Caco-2 intestinal epithelial cells results in a partial EMT. This was observed both at a cellular level, in terms of the effects on morphology, cell-cell junctions and cytoskeletal distribution and at the molecular level, in terms of changes in gene expression. The primary epithelial feature lost in *Neo1-*depleted epithelia is the circumferential ZA. In contrast the tight junctions appeared unaffected. Substantial changes to the F-Actin cytoskeleton were seen in basal regions of the cells, where the stress-fibres were replaced with extensive lamellipodial ruffles, characteristic of migratory mesenchymal cells. Thus while the most apical parts of *Neo1-*depleted epithelia maintain their tight junction cell-cell connections, in the basal regions below cells appear migratory. We speculate that this underlying protrusive activity causes epithelial cells within the islands to move laterally resulting in the sliding phenotype we observed. In addition, at the edges of epithelial islands, loss of *Neo1* resulted in an increase in the proportion of cells with a migratory mesenchymal morphology. Our results corroborate other studies where loss of *Neo1* has been associated with increased rates of cell migration in MDA-MB-231 human breast cancer cells^32^. They are also consistent with experiments in which downregulation of Neo and DCC via the activity of serine proteases increased the motility of MCF-7 cells in a wound healing assay^33^.

Loss of ZAs is a central feature of EMT and the transcriptional profile of *Neo1-*depleted cells exhibited an EMT-like signature with upregulation of ECM components (*FN1*), integrin ECM-receptors (*ITGA2*, *ITGA5*, *ITGB1*) and ECM regulators like *MMP1*. Many of the upregulated genes could also be associated with a more metastatic phenotype. For example, de Graauw *et al*.^34^ showed that *ANXA1* expression was associated with a highly invasive basal-like breast cancer subtype in a panel of breast cancer cell types^34^. Other studies have also suggested a possible role for ANXA1 expression and tumour cell migration in pancreatic and prostate cancers^35^. L1CAM augments colon cancer cell metastasis by activating NF-kb signalling without inducing a change in the classical EMT and cancer stem cell markers^36^. Our western blot analysis showed minimal changes in the total cellular E-Cad and ZO-1 levels but significant upregulation of FN1. Considering that we see some of the EMT markers going up (FN1, MMP1) after *Neo1* knockdown, stable E-Cad levels and decreased levels of *KRT 8* and *KRT 18* (Supplementary Fig. S8), we think that in our system the cells are displaying a hybrid/partial EMT phenotype associated with a “stemness” of the cell population and a more aggressive metastatic behaviour^37^.

In addition to changes in the ZA and F-Actin, there were distinct effects on the MT cytoskeleton. In *Neo1-*depleted cells the MT array was less densely packed and EB1 plus-end comets were longer and moved more quickly. The relationship between ZAs and MTs is complex and can involve interactions with both plus and minus MT-ends. AJs can interact with the plus end of MTs at the cell cortex, and if this binding is disrupted then cells fail to concentrate E-Cad at the ZA^38^. AJs can also regulate microtubules. The minus-end directed motor dynein exhibits F-Actin dependent localisation to AJs and can directly bind to beta-catenin^39^, where it is thought to recruit and tether MTs, thus mediating crosstalk between actin and MTs at the cell cortex. Similarly, E-Cad and N-Cad mediated signalling in cell-cell junctions has been shown to stabilize MTs^40^. This study showed that wild type CHO cells have densely packed radial MT arrays while cells which lack centrosomes have sparsely populated MTs. However, cells that were devoid of centrosomes but which maintained their cell-cell junctions showed MT densities equivalent to wild type cells suggesting involvement of cell-cell junction mediated signalling in maintaining MT levels. Another study has also shown that in fibroblasts devoid of centrosomes, the MTs become sparsely populated due to the depolymerization of minus ends^41^. Thus, in our system disruption of AJs and redistribution of E-Cad may serve to depolymerize minus ends of MTs and reduce microtubule density.

*Neo1* depletion also affected EB1 and plus-end microtubule dynamics, which is known to play a key role in cell motility. Phosphorylation of EB1 at Serine 155 residue has been found to promote cancer cell migration and proliferation in a study conducted by Le Grand *et al*.^42^. Ser155 is in the linker region of EB1, which mediates the microtubule binding ability of these molecules by promoting the calponin homology domain binding. EB1 is also crucial to maintain a balance between formation of filopodia and lamellipodia. Cells lacking EB1 protein were shown to have more filopodial extensions which led to a decrease in overall cell migration velocity^43^. Further work will be needed to understand the relationship between the loss of ZAs, the decrease in microtubule density and the increase in microtubule dynamics.

In addition to the induction of partial-EMT, loss of *Neo1* caused an extensive transcriptional response consistent with a defence against pathogens. *Neo1-*depleted cells upregulated genes with anti-bacterial, anti-viral and anti-fungal properties as well as those associated with blood coagulation and inflammation. Similar transcriptomic profiles have been seen in other studies on colorectal cancer tissues. Lin *et al*.^44^ sequenced 48 primary colorectal cancer and 20 liver metastatic samples and found that genes associated with inflammatory pathways and tissue remodelling were upregulated while those related to oxidative phosphorylation and proliferation were downregulated^44^.

While *Neo1-*depleted cells exhibited an increased partial EMT signature genes associated with oxidative phosphorylation were downregulated. This anti-correlation of EMT and oxidative phosphorylation appears to be a recurring feature of cancer metastasis. A systems analysis conducted by Gaude *et al*.^45^ on 20 solid tumour types showed that downregulation of mitochondrial genes associated with oxidative phosphorylation correlated with a higher expression of EMT genes in 9/15 (60%) cancers and poor clinical outcome^45^. Notably, they also found a negative correlation between genes associated with oxidative phosphorylation and those associated with EMT in 19/20 cancer types. Interestingly in our study, the majority of the oxidative phosphorylation related genes that were downregulated encoded different subunits of NADPH dehydrogenase complex I which may suggest partial inhibition of the electron transport chain. Partial inactivation of complex I leads to enhanced ROS production and it has been associated with enhanced metastatic potential of tumour cells^46^. It is also possible that these cancer cells with *Neo1-*depletion are exhibiting a form of the Warburg effect in which higher levels of aerobic glycolysis occur^47^. This might correlate with increases in NOX1 since it has been shown that the production of NAD+ via NOX is critical for cells with increased aerobic glycolysis^48^.

In summary, our interpretation of these observations is that the loss of Neo leads to loss of ZA integrity, which is interpreted as damage to the intestinal epithelial barrier function. As a consequence, cells increase their ability for epithelial migration, while maintaining their tight junction barrier function, to close any gaps/lesions in the epithelium. They also guard against pathogens by secreting proteinases, lyzozymes and interferons, and increasing ROS production, and guard against blood loss by increasing coagulation factors.

In conclusion, Loss of *Neo1* results in changes in gene expression and cellular morphology indicative of a partial EMT. Cells maintain their epithelial tight junction connections but exhibit a more mesenchymal, migratory morphology and substantial changes to the cytoskeleton with a reduction in F-Actin stress-fibres and fewer, but more dynamic microtubules. Transcriptomic analysis shows that in addition to the cells shifting towards a partial EMT, there is also a more general wound healing response with upregulation of anti-microbials and inflammatory cytokines.

## Methods

### Cell Culture

All experiments were performed using human colorectal cancer cells (Caco-2) which were a kind gift from Dr Michelle Palmieri (Walter and Eliza Hall Institute, Melbourne, Australia). Cells were cultured in DMEM medium (Sigma-Aldrich, St Louis, MO) supplemented with 10% foetal bovine serum (FBS) (Bovogen Biologicals, Melbourne, Australia), 2mM L-Glutamine and 1% penicillin-streptomycin solution (both from Sigma) and were maintained at 37 °C and 5% CO_2_ in a humidified incubator. Cells were passaged at 60–70% confluency and cells less than 20 passages were used for all experiments. Cells were routinely tested for mycoplasma and were found mycoplasma negative. All other general chemicals have been purchased from Sigma unless otherwise mentioned.

### siRNAs, plasmids and transfections

Pre-designed and validated negative control-siRNA and human *Neo1* siRNA duplexes were purchased from Ambion (Thermo Fisher Scientific, Waltham, MA). Caco-2 cells were transfected either, 24 h after seeding-Post-transfected or at the time of cell seeding – Co-transfected with 75 nM of siRNA (control or *Neo1*) using Lipofectamine 2000 (Invitrogen) according to the manufacturer’s instructions. The effects of *Neo1* knockdown were analysed 24–120 hr post-transfections. C-terminal GFP-tagged human EB1 plasmid (Cat No. 39299) was purchased from Addgene (Cambridge, MA).

### Antibodies (Immunocytochemistry-ICC, Western blotting-WB)

Primary antibodies – EB1 (ICC-1:250, WB-1:500), GAPDH (WB-1:2500), MMP-1 (ICC-1:250), alpha-tubulin (ICC-1:500) all purchased from Abcam, Cambridge, UK. Fibronectin (ICC-1:100, WB-1:1000, Developmental Studies Hybridoma Bank, Iowa city, IO), NEO1 (ICC-1:50, WB-1:250, Santa Cruz Biotechnology, Dallas, TX), ZO-1 (ICC-1:350, WB-1:2500, Thermo Fisher Scientific), E-Cad (ICC-1:250, WB-1:2500, BD Biosciences), L1CAM (ICC-1:1000, a kind gift from Prof. Heather Young, University of Melbourne). Alexa Fluor (AF) conjugated secondary antibodies for ICC – Donkey anti-goat AF 488, Donkey anti-mouse AF 546 and 647, Goat anti-rat AF 488 and 546, Goat-anti rabbit AF 488. All the secondary antibodies for ICC were used at a dilution of 1:500, were highly cross-adsorbed and purchased from Molecular probes. Horse-radish peroxidase (HRP) conjugated secondary antibodies for WB- Goat anti-mouse HRP, Goat anti-rat HRP, Goat anti-rabbit HRP, Donkey anti-goat HRP. All HRP conjugated antibodies were used at a dilution of 1:5000 and were purchased from Jackson Labs (Bar Harbor, ME). Rhodamine Phalloidin (1:250, Cytoskeleton Inc, Denver, CO), 4′,6-diamidino-2-phenylindole or DAPI (1:1000, Thermo Fisher Scientific).

### AQueous One metabolic assay

Metabolic activity of the cells was measured using CellTiter AQueous One Solution (Promega) according to the manufacturer’s instructions. This assay measures the cells ability to reduce a tetrazolium compound to a soluble formazan product, likely through NADPH or NADH produced by dehydrogenases in metabolically active cells. Briefly, Caco-2 cells were seeded in 24-well plates (Nunc) at a density of 10 × 10^3^ cells per well, co-transfected with either Control or *Neo1*-siRNAs. After 72 h in culture, 100 µl of CellTiter AQueous One Solution was added and cells were incubated at 37 °C with 5% CO_2_ for 4 h. The absorbance of each well was then measured at 490 nm using a plate reader (EnSpire Multimode plate reader, Perkin Elmer). Caco-2 cells without transfection reagent were used as a positive control. Blank DMEM medium served as a negative control and relative cell numbers were represented as absorbance per well. Previous experiments indicated the cell numbers obtained were in the linear range of an AQueous One standard curve.

### Annexin-V FITC apoptosis assay

Apoptosis assay was performed on control-siRNA and *Neo1*-siRNA treated cells after day 3 of co-transfection using Annexin-V apoptosis kit (Molecular Probes, Thermo Fisher Scientific). Briefly, 1 × 10^5^ cells/well were seeded in a 6-well plate and co-transfected with Control or *Neo1*-siRNA. After 72 hr, cells were washed in cold PBS, trypsinised, centrifuged and were resuspended in 200 ul of Annexin-binding buffer (1x). 2.5 μl of annexin-V FITC and 1 μl of propidium iodide (PI) was then added to the cells respectively and incubated for 15 min at RT. After the incubation, 200 μl of annexin binding buffer was added to the cells and fluorescence emission was then read at 530 nm using a BD LSR Fortessa flow cytometer (BD Biosciences, NJ).

### Cell lysis and Western Blotting

Caco-2 cells were grown in 6-well plates (Nunc) and transfected with control or *Neo1*-siRNA. Cell lysates were prepared 48 h after transfection using KALB lysis buffer (150 mM NaCl, 50 mM Tris (base), pH 7.4, 1 mM EDTA, 1% Triton X-100 and 10% glycerol) and 1x complete protease inhibitor cocktail (Roche, Mannheim, Germany). Briefly, 250 μl of ice cold lysis buffer was dispensed into each well and the plate was incubated on ice for 5 min. The cell suspension was then transferred to a 1.5 ml Eppendorf tube and centrifuged at 14000 rpm for 15 min at 4 °C. The supernatant was collected in a fresh tube and protein concentration was measured using a pierce BCA protein assay kit (Pierce Biotechnology, Rockford, IL).

For western blots (WB), cell lysates (20 μg) were separated by SDS-PAGE on a 4–15% Mini-PROTEAN TGX precast gradient gel (80 V, 90 min) (Bio-Rad, Hercules, CA) and proteins were transferred to an Immun-blot PVDF membrane (35 V, 90 min) (Bio-Rad). The membrane was then blocked in 5% skimmed milk/0.1% Tween-20/PBS (PBST) for 1 hr at RT and incubated with primary antibodies for either overnight at 4 °C or 2 h at RT. Membrane was washed 4 times with PBST and incubated in HRP-conjugated secondary antibodies for 3 h at RT. Membrane was washed and incubated in Clarity enhanced chemi-luminescence western blotting substrate (BioRad) for 5 min and imaged using a ChemiDoc MP Imager (BioRad).

### Immunofluorescence and confocal microscopy

Caco-2 cells (5 × 10^4^/cm^2^) were seeded on iBiDi μ-dishes (DKSH, Switzerland), transfected with control-siRNA or *Neo1*-siRNA and cultured for 1, 3 or 5 days after transfection. Post-transfection, cells were fixed using 3.7% paraformaldehyde/PBS for 10 min or ice-cold methanol (100%) for 5 min, permeabilized in 0.1% TX-100/PBS for 8 min and incubated in blocking solution (10% FCS in PBS) for 2 hr at RT. Cells were then incubated with respective primary antibodies for 3 hr at RT or overnight at 4 °C, washed in PBS (3 times) and incubated in Alexa fluor conjugated-secondary antibodies for 2 hr at RT. Cells were then washed with PBS, mounted in 80% glycerol/PBS and imaged using an Olympus FV1000 or a Nikon A1R confocal microscope. All images were acquired at 1024-pixel resolution with either A 40x/60x water or oil objective and similar settings of the microscope.

### EB1 comet assay, MT density and cell movement analysis

EB1 comet shapes were quantified using ImageJ (version 1.51 h, https://imagej.nih.gov/ij/). Control and *Neo1-*depleted Caco-2 cells were cultured, fixed and immunostained for ZO-1 and EB1 and image stacks collected at 0.5 μm intervals. Maximum intensity projections were calculated and cell outlines derived from the ZO-1 channel. To analyse EB1 comets, the resolution of images was resized 3-fold, and a rolling-ball background subtraction applied. EB1 comets were determined using the Analyze Particles function and the average aspect ratio calculated for each cellular region. Aspect ratio (AR) was used as a proxy for comet length since the comet width was relatively constant (macro available on request).

For live imaging (time-lapse) of human EB1-GFP (500 ng) transiently transfected control and *Neo1*-siRNA treated cells, images were acquired using a Leica TCS SP5 confocal microscope (Leica microsystems, Wetzlar, Germany) with a 63x oil objective. Images of EB1 comets were taken at an interval of every 2 sec for a total of 90 sec. EB1 speed analysis was done using the spot and track function in Imaris software ver. 8.4.1 (Bitplane, Zurich, Switzerland).

For MT density analysis, maximum intensity projections of the 1 μm z-stacks were calculated and a rolling-ball background subtraction applied. MTs were detected using the ImageJ “Ridge Detection” plugin (v1.4.0) (Thorsten Wagner and Mark Hiner)^49^. To convert the resultant linear ROI shapes to a measure of MT density, they were rendered (drawn) as white lines onto a black image and the average intensity calculated (macro available on request).

To quantify the lateral movement of cells, time-lapse sequences were first processed with an Image Stabilizer plugin (K. Li, http://www.cs.cmu.edu/~kangli/code/Image_Stabilizer.html), and then temporally smoothed by calculating a running average over 50 time-frames (Supplementary Movie S1). This served to average out fast, jiggling, movements of features such as organelles, leaving only the steady lateral movements of cell-cell junctions and net movement of organelles and other visible features. Regions were chosen to lie entirely within epithelial islands (i.e. excluding peripheral edges) and these were then time-sliced in both x and y directions and the resultant kymograph images processed to detect ridges. These linear traces were then analysed to determine the Feret angle which yielded the absolute angle away from the vertical, and these were then averaged for each region (Supplementary Fig. S9).

### RNA-seq analysis of Caco-2 cells

Total RNA was isolated from cells using a RNeasy mini kit (Qiagen, Hilden, Germany). RNA integrity was assessed using a Nanodrop spectrophotometer (Thermo Scientific) and an Agilent 2100 Bioanalyser and all samples were found to have RIN values of ≥9.9 except one sample where RIN value was 7.9. cDNA was synthesised from extracted RNA and library prepared using the Illumina TruSeq library preparation kit (San Diego, CA) and sequenced on an Illumina Hiseq. 2500 platform running in 100 bp single-end mode.

In total, for all the samples, 275 million reads passed the quality control. Individual samples yielded a range of 21–25 million reads. Sequencing reads were aligned to the Ensembl human genome GRCh38 (ftp://ftp.ensembl.org/pub/release-85/fasta/homo_sapiens/dna/) using Tophat (v2.1.1) built with Bowtie (ver. 2.2.2) with default parameters^50^. For each sample, 92.0–93.2% of the reads mapped to the genome. The reads mapped to individual genes were then counted using Cuffquant using the annotation file v.21 for every sample. The count files for the different samples in cxb format were then merge using Cuffdiff.

Gene-based read counts were analysed using the iDEP (v web application http://ge-lab.org/idep/). Genes with less than 0.5 counts per million (CPM) in every sample were discarded. 18,282 genes were included for further analysis. Gene expression level was normalised using a log2(CPM + c) transform with a pseudo count c = 4 implemented in the edgeR package (v3.20.9) and missing values were imputed using gene median for clustering and principal component analysis (PCA). Differentially expressed genes were analysed using a linear-mixed model as factoring the effect of *Neo1* siRNA-treated Caco-2 vs. control siRNA treated cells as experimental factor as implemented in the limma package (v3.34.9). K-means clustering and pathway enrichment analysis were performed using iDEP v0.73.

### Quantitative real-time PCR (qPCR)

RNA was isolated from the Caco-2 cells using RNeasy mini kit (Qiagen) according to the manufacturer’s instructions. RNA concentration and purity were assessed using a Nanodrop spectrophotometer and all samples were found to have A260/280 ratio of >2.0. Reverse transcription was performed on 1.0 μg of RNA using the Tetro cDNA synthesis kit (Bioline, Alexandria, Australia). qPCR reactions were performed using SsoAdvanced Universal SYBR Green Super mix (Bio-Rad), with triplicate reactions containing 5 μl SYBR Green mix, 1 μl of forward/reverse primer (0.5 μM), 2 μl of template DNA and 2 μl of RNase free water. Reactions were performed on a CFX96 Touch Real-Time PCR system (Bio-Rad) with a 96-well block using the following conditions: denaturation at 95 °C for 2 min, 40 cycles of denaturation at 95 °C for 5 sec, annealing at 60 °C for 10 sec, extension at 72 °C for 20 sec followed by a melt step ranging from 55 °C to 95 °C. Primers were selected for six genes of interest (GOI)-Carcinoembryonic antigen-related cell adhesion molecule (*CEACAM1*), Annexin A1 (*ANXA1*), NADPH Oxidase I (*NOX1*), L1 cell adhesion molecule (*L1CAM*), Keratin 8 (*KRT8)* and Keratin 18 (*KRT18*) and three reference genes (RG): Tata binding protein (*TBP*), beta-actin (*ACTB*) and Glyceraldehyde-3-phosphate-dehydrogenase (*GAPDH*) using the Primer bank database (https://pga.mgh.harvard.edu) and were purchased from Integrated DNA Technologies (Singapore). For primer sequences refer to Table 1. Of all three reference genes tested, *TBP* was found to have the most stable expression for control and *Neo1-siRNA* treated samples and hence was used for normalization. Expression levels for six GOI were normalized to *TBP* expression values and fold change was calculated using the 2^−deltadelta^ Ct method^51^ with relative to control-siRNA. Mean ± S.E. of 6 biological replicates are shown from two independent experiments. Student’s t-test (two-tailed) was performed and it was considered significant with a p-value < 0.05.

**Table 1:** List of primers used for qPCR.

Gene name	Sequence 5′ – 3′ (FP, RP)	NCBI reference no.	Primer bank ID	BP
TBP	CCACTCACAGACTCTCACAAC	6908	285026518c1	127
	CTGCGGTACAATCCCAGAACT			
GAPDH	ACAACTTTGGTATCGTGGAAGG	2597	378404907c2	101
	GCCATCACGCCACAGTTTC			
ACTB	CATGTACGTTGCTATCCAGGC	60	4501885a1	250
	CTCCTTAATGTCACGCACGAT			
KRT18	GTTGACCGTGGAGGTAGATGC	3875	40354194c3	86
	GAGCCAGCTCGTCATATTGGG			
KRT8	CAGAAGTCCTACAAGGTGTCCA	3856	372466576c1	194
	CTCTGGTTGACCGTAACTGCG			
L1CAM	CCGACAACCACTCAGACTACA	3897	221316755c2	83
	CCGGAGGTCAATGGGTTCC			
CEACAM1	TGCTCTGATAGCAGTAGCCCT	634	329112546c1	56
	TGCCGGTCTTCCCGAAATG			
ANXA1	CTAAGCGAAACAATGCACAGC	301	4502100c2	111
	CCTCCTCAAGGTGACCTGTAA			
NOX1	GCACACCTGTTTAACTTTGACTG	27035	148536872c3	129
	GGACTGGATGGGATTTAGCCA			

### Statistical analysis

All the statistical analysis was done using the software GraphPad Prism ver 6.0 (La Jolla, CA). The data from three to four independent replicates per data point was collected from at least two independent experiments and represented as means ± standard error unless otherwise mentioned. Statistical significance was calculated using a non-parametric student’s t-test (two-sided) with p < 0.05 considered as significant.

## Supplementary information

Supplementary Information

Supplementary Movie S1

Supplementary Movie S2

Supplementary Movie S3

Supplementary Movie S4

Supplementary Movie S5

Supplementary Table S1

Supplementary Table S2

Supplementary Table S3

## Data Availability

All data generated or analysed during this study are included in this published article and its supplementary files.
