# Supplementary material for: Loss of Neogenin1 in human colorectal carcinoma cells causes a partial EMT and wound-healing response
Source: Sci Rep. 2019 Mar 11;9:4110. doi: 10.1038/s41598-019-40886-y (PMC6411945; doi:10.1038/s41598-019-40886-y)

Supplementary information

Loss of *Neogenin1* in human colorectal carcinoma cells causes a partial EMT and wound-healing response

Vishal Chaturvedi[1], Alexandre Fournier-Level[1], Helen M Cooper[2], Michael J. Murray[1]

[1] School of Biosciences, The University of Melbourne, Melbourne, Victoria 3010, Australia

[2] The University of Queensland, Queensland Brain Institute, Brisbane, Queensland 4072, Australia

Corresponding author: murraym@unimelb.edu.au. Phone: +61383447139

Supplementary Figure S1 *Neo1* knockdown in Caco-2 cells was confirmed by Western blot analysis. Representative blot with three biological replicates from one experiment. The boxed region has been shown in Figure 1. Same blot has been used for GAPDH probing after being stripped using a mild stripping buffer (Abcam)

Supplementary Figure S2 No significant change in E-Cad protein levels after *Neo1* knockdown. Each band represents cell lysate proteins from a biological replicate from three independent experiments. The boxed region has been shown in Figure 1 and the blot has been scanned at high and low contrast. Same blot has been used for GAPDH probing after being stripped using a mild stripping buffer (Abcam)

Supplementary Figure S3 Western blot for ZO-1 in control and *Neo1*-siRNA treated cells. The boxed region has been shown in Figure 1 and the blot has been scanned at high and low contrast. Blot outline has been marked using dotted lines

Supplementary Figure S4 **a.** DLD-1, RKO, and SW480 cells treated with control or *Neo1*-siRNA and stained for F-Actin and immunostained for E-Cad. siRNA was transfected into 1-day old epithelia and cells stained after 2 days. Scale bar-20μm. **b.** RT-qPCR of *Neo* relative to the reference gene (*TBP*) in different CRC lines. Error bars show SEM; p-values based on two-tailed student's t-test relative to Caco-2 levels.

Supplementary Figure S5 Significant upregulation of fibronectin protein levels after *Neo1* knockdown. Each band represents cell lysate proteins from a biological replicate from three independent experiments. The boxed region has been shown in Figure 4 and the blot has been scanned at high and low contrast. Same blot has been used for GAPDH probing after being stripped using a mild stripping buffer (Abcam).

Supplementary Figure S6 Quantification of the perimeter fraction of mesenchymal cells per island of control and Neo1 siRNA treated cells. $P<0.0001$

Supplementary Figure S7 No significant change in EB1 protein levels after *Neo1* knockdown. Each band represents cell lysate proteins from a biological replicate from one experiment. The boxed region has been shown in Figure 6 and the blot has been scanned at high and low contrast.

Supplementary Figure S8 RT-qPCR of control and *Neo1* knockdown post-transfected Caco-2 cells shows significant downregulation of *KRT8* and *KRT18* in *Neo1* knockdown cells suggesting partial EMT. Data represent mean ± SEM of 6 biological replicates, $p=0.04$ and $p=0.01$ respectively (two-tailed student's t-test)

Supplementary Figure S9 Kymograph analysis of control and *Neo1*-knockdown Caco-2 cells revealed an increase in the angle of movement of internal cellular features in *Neo1* knockdown cells. Time-lapse movie images (i) captured at 15 sec intervals (ii) were resliced in the time-dimension to create kymographs (iii), which were then analysed using ridge detection (iv) to detect lateral movement of internal features and the Feret angle for each ridge determined (v). See Materials and Methods for details.

Supplementary Movie S1 - Time-lapse imaging of control and *Neo1* knockdown cells was performed and epithelial islands were imaged for a total of 30 min with an image every 15 sec. To quantify the lateral movement of cells, collected sequences were processed with an Image Stabilizer plugin (K. Li, http://www.cs.cmu.edu/~kangli/code/Image_Stabilizer.html), and then temporally smoothed by calculating a running average over 50 time-frames. The split panel shows an example of time lapse movie before and after smoothing.

Supplementary Movie S2 - Live imaging of Neo1 siRNA transfected Caco2 epithelial islands showing cell migration. Images were taken at every 15 sec for a total of 30 min

Supplementary Movie S3 - Live imaging of control siRNA transfected Caco2 epithelial islands showing stable islands. Images were taken at every 15 sec for a total of 30 min

Supplementary Movie S4 -Time lapse imaging of EB1 comets in *Neo1* siRNA transfected Caco2 cells showed faster comets. Cells were transfected with *Neo1* siRNA and EB1-GFP plasmid and 24 h later were imaged over a period of 2 min with an image taken every 2 sec

Supplementary Movie S5 - Time lapse imaging of EB1 comets in control siRNA transfected Caco2 cells. Cells were transfected with control siRNA and EB1-GFP plasmid and 24 h later were imaged over a period of 2 min with an image taken every 2 sec

Supplementary Table S1 – Table of genes that were significantly upregulated and downregulated in Pathway enrichment analysis for *Neo1* siRNA transfected cells at day 2 post-transfection. Pathway enrichment analysis was performed using iDEP v0.73.

Supplementary Table S2 - Table of genes that were significantly upregulated and downregulated in Pathway enrichment analysis in *Neo1* siRNA transfected cells at day 5 after co-transfection. Pathway enrichment analysis was performed using iDEP v0.73.

Supplementary Table S3 – Table of genes showing K-means cluster pathway analysis for upregulated and downregulated genes in *Neo1* siRNA co-transfected cells

Figure S1

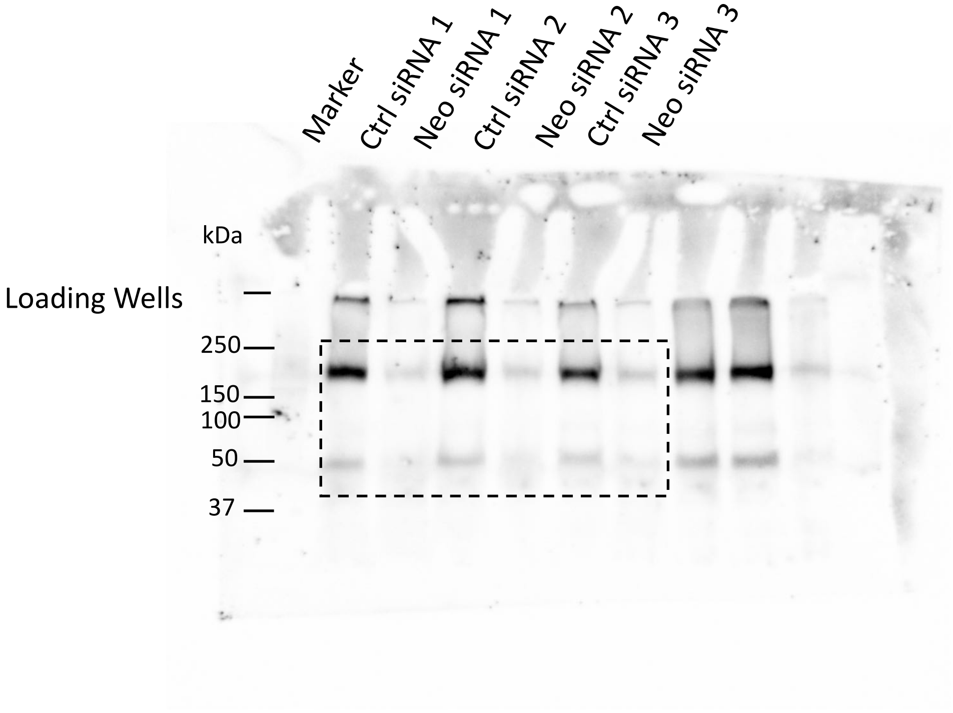

Neogenin western blot

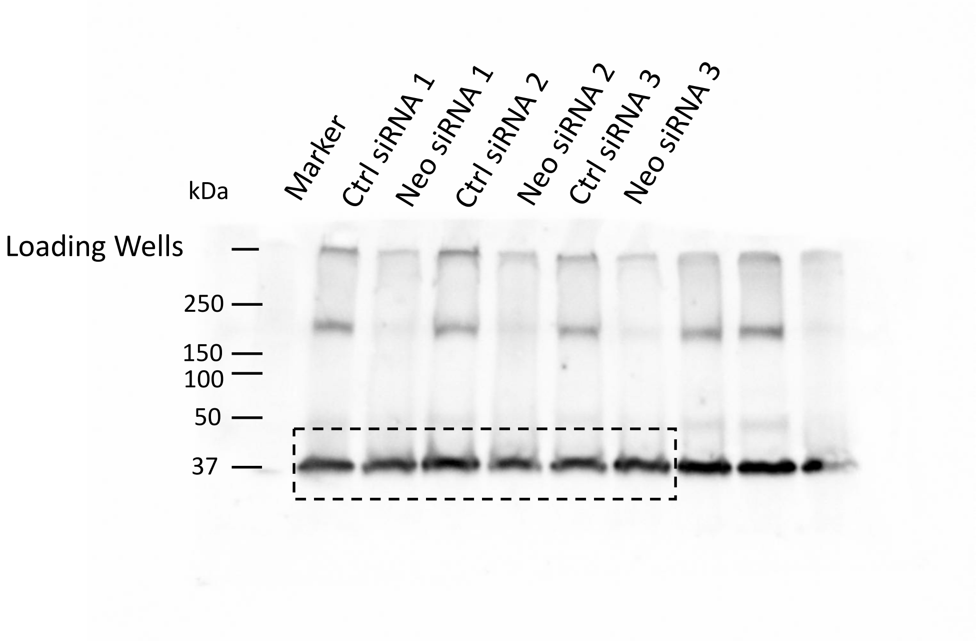

GAPDH western blot

Figure S2

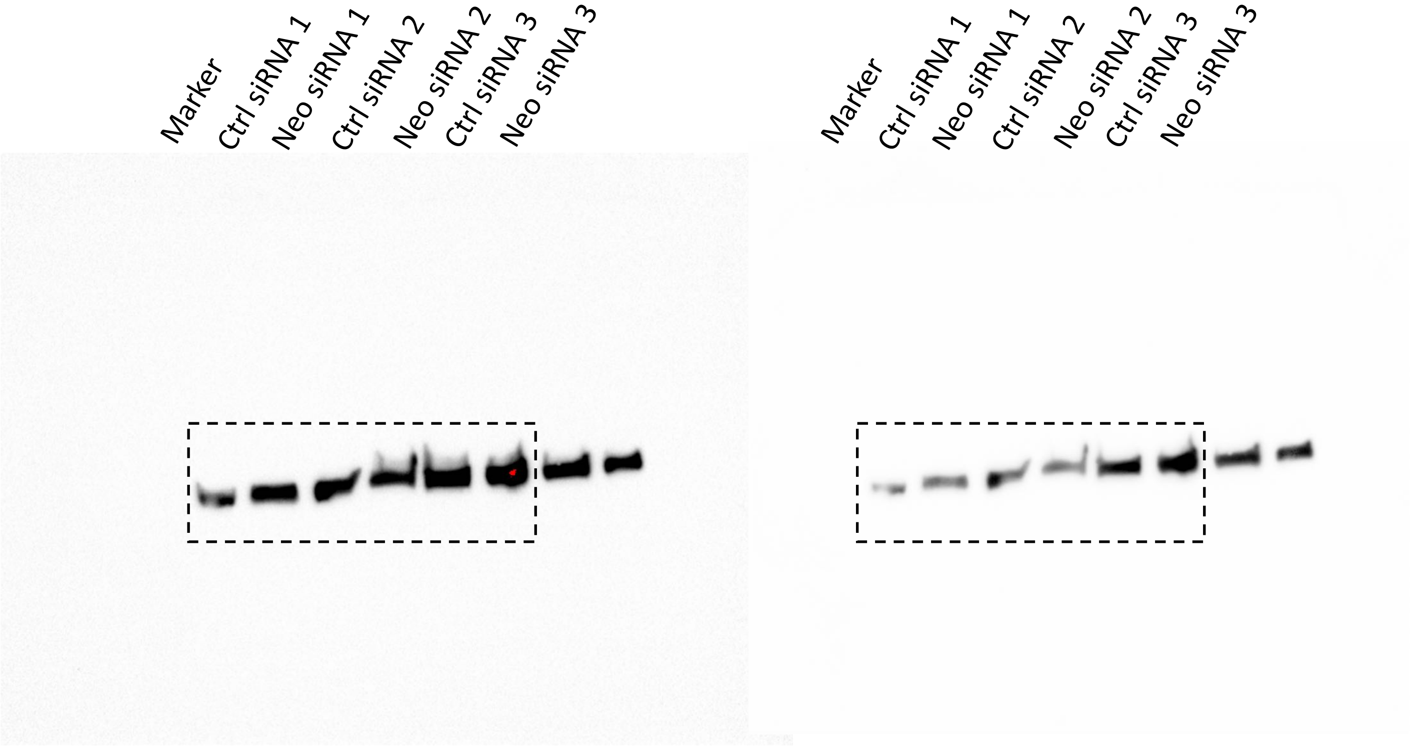

E-Cad blot

High contrast

Low contrast

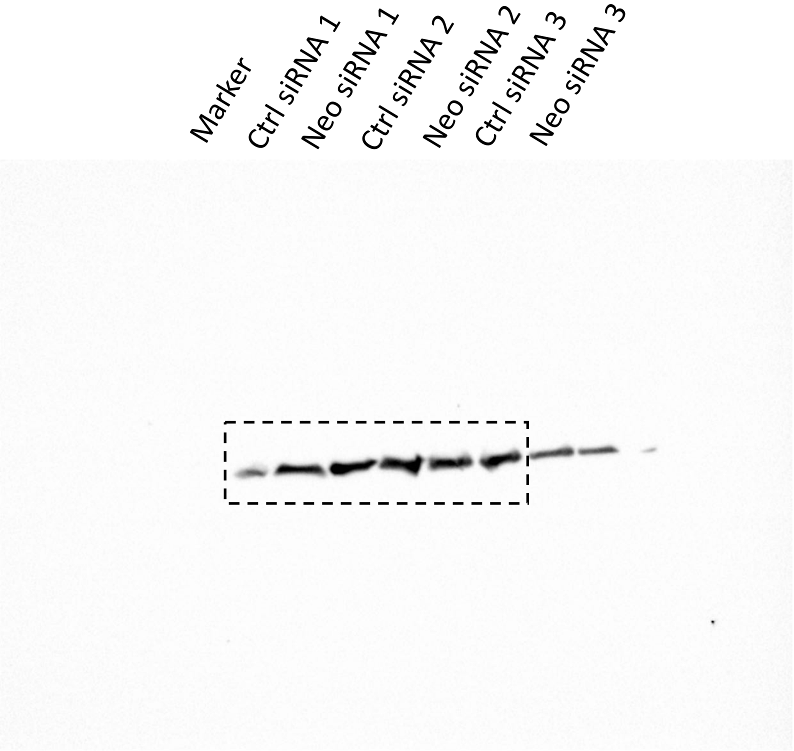

GAPDH blot

Figure S3

ZO-1 blot

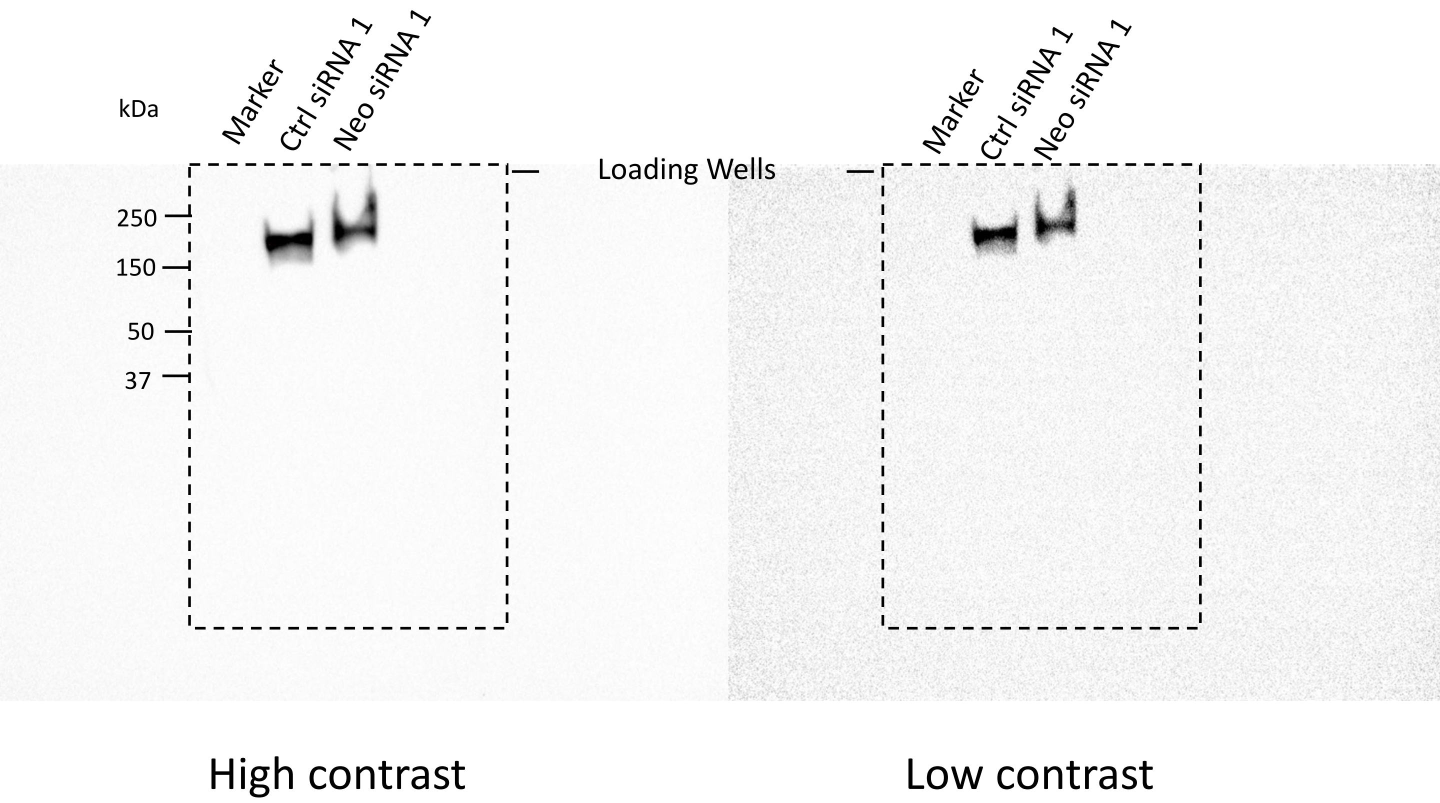

Figure S4

a

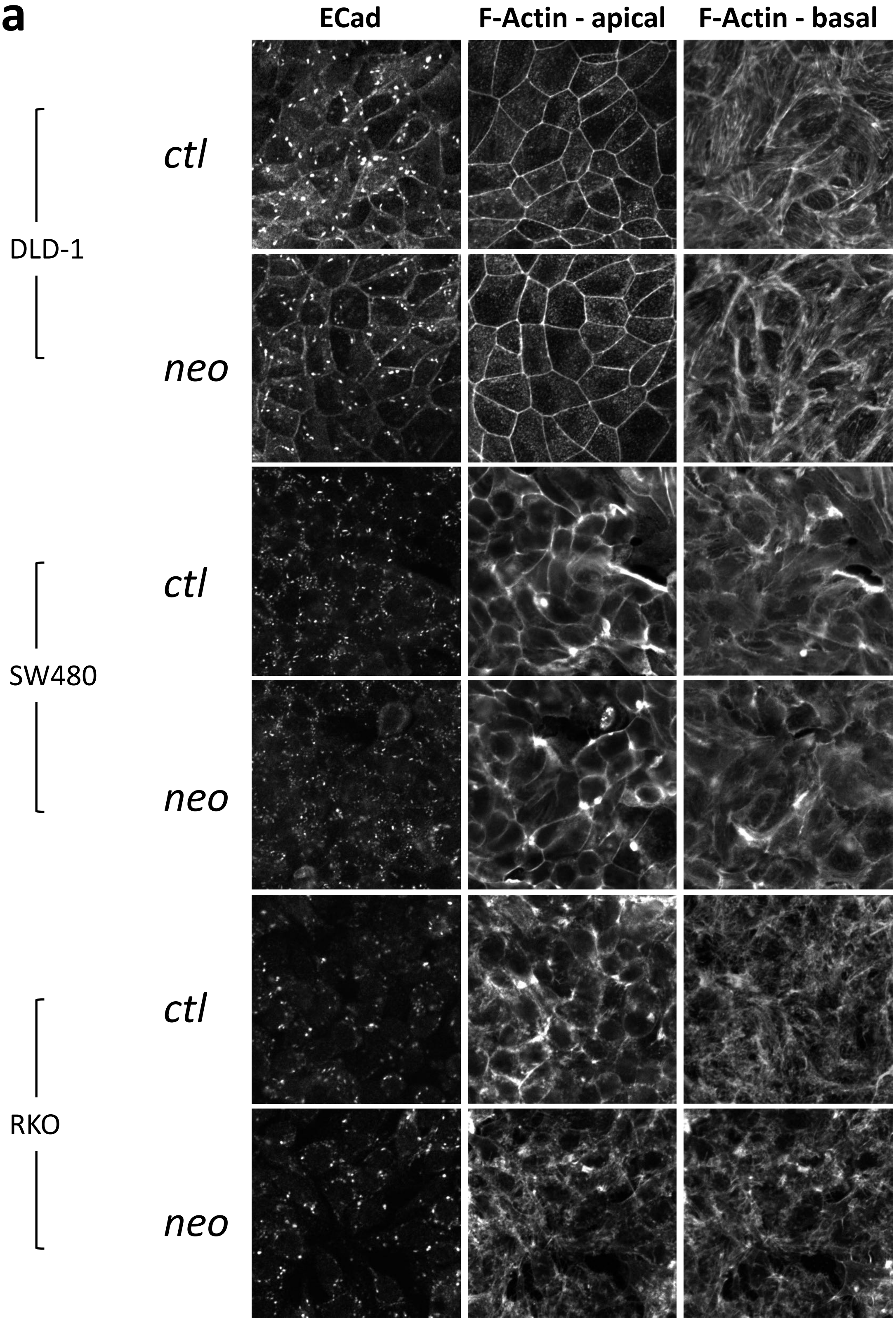
ECad
F-Actin - apical
F-Actin - basal
DLD-1
ctl
neo
SW480
ctl
neo
RKO
ctl
neo

b

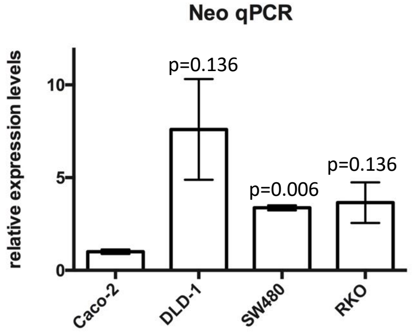
Neo qPCR
relative expression levels
10
5
0
p=0.136
p=0.006
p=0.136
Caco-2
DLD-1
SW480
RKO

Figure S5

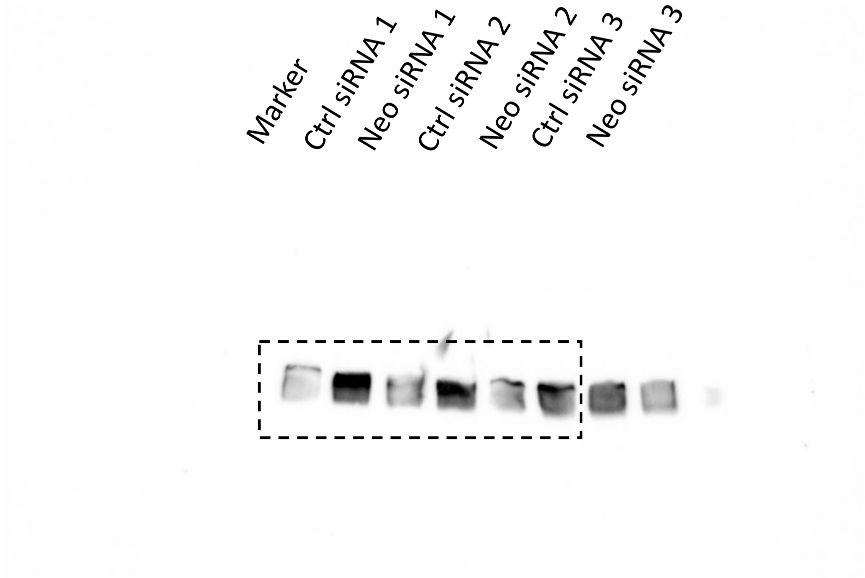

Fibronectin blot

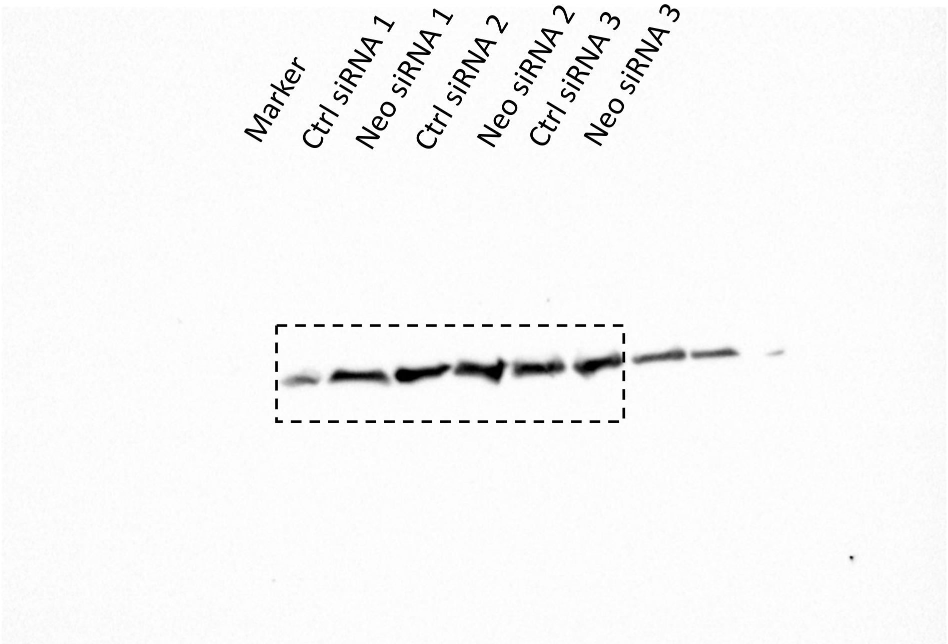

GAPDH blot

Figure S6

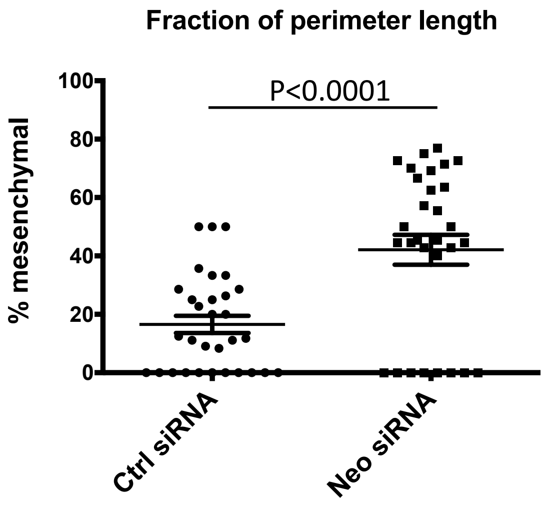
Fraction of perimeter length
P<0.0001
% mesenchymal
100
80
60
40
20
0
Ctrl siRNA
Neo siRNA

Figure S7

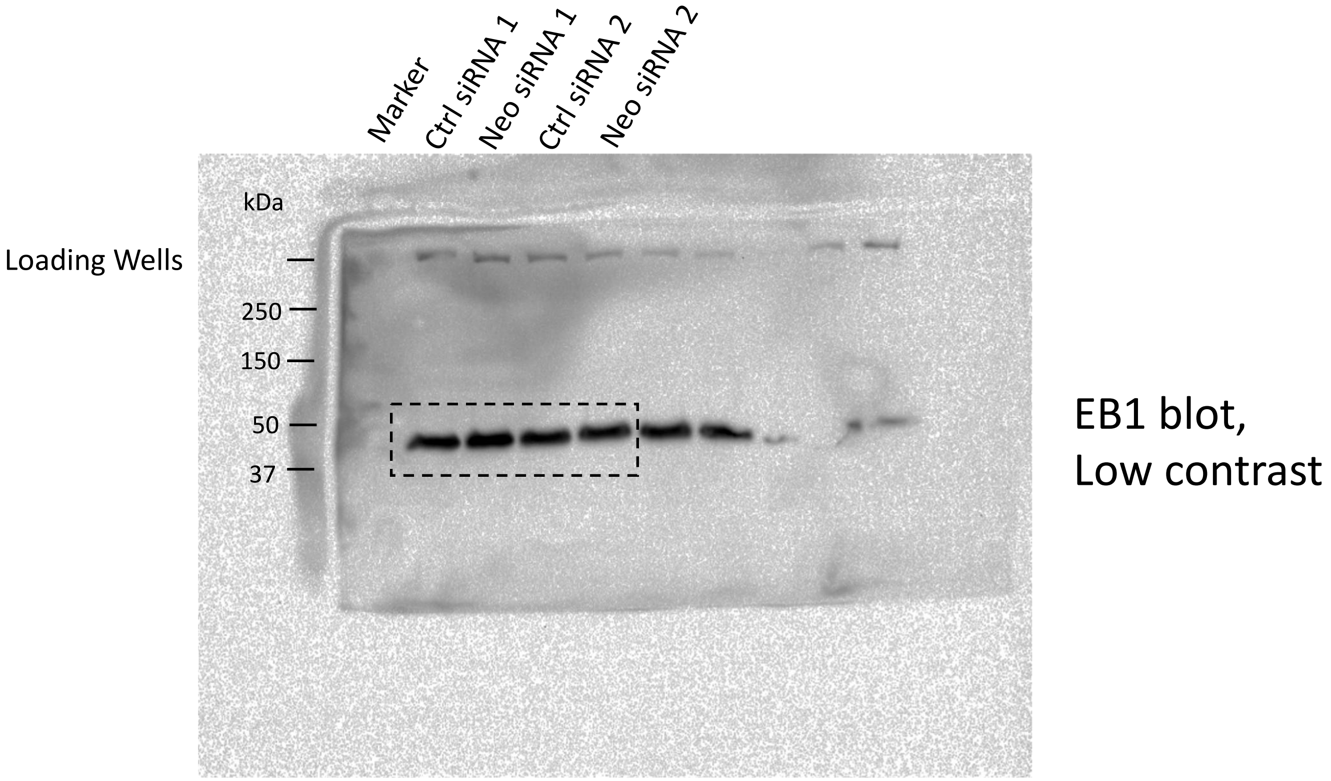
Marker
Ctrl siRNA 1
Neo siRNA 1
Ctrl siRNA 2
Neo siRNA 2
kDa
Loading Wells
250
150
50
37
EB1 blot,
Low contrast

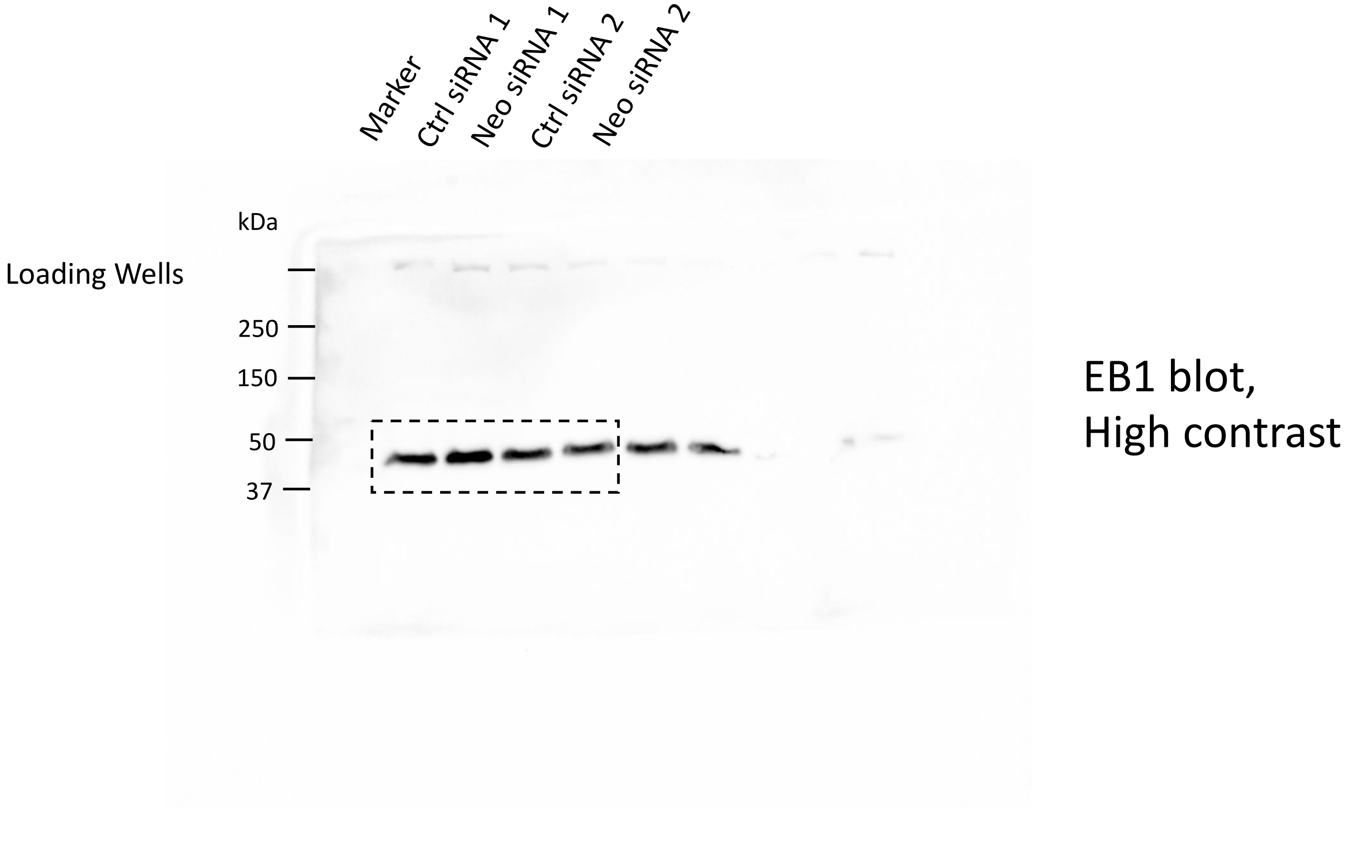
Marker
Ctrl siRNA 1
Neo siRNA 1
Ctrl siRNA 2
Neo siRNA 2
kDa
Loading Wells
250
150
50
37
EB1 blot,
High contrast

Figure S8

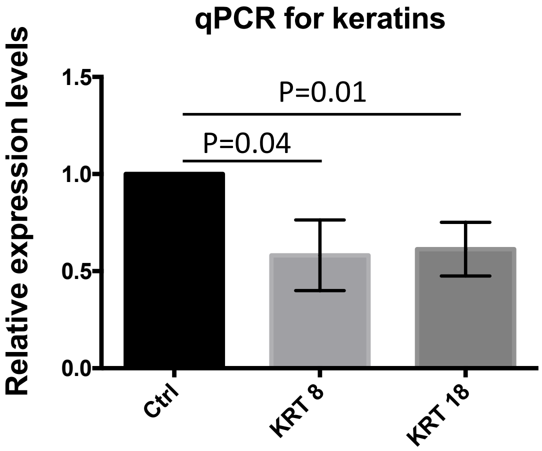
qPCR for keratins
Relative expression levels
1.5
1.0
0.5
0.0
P=0.01
P=0.04
Ctrl
KRT 8
KRT 18

Figure S9

Kymograph construction

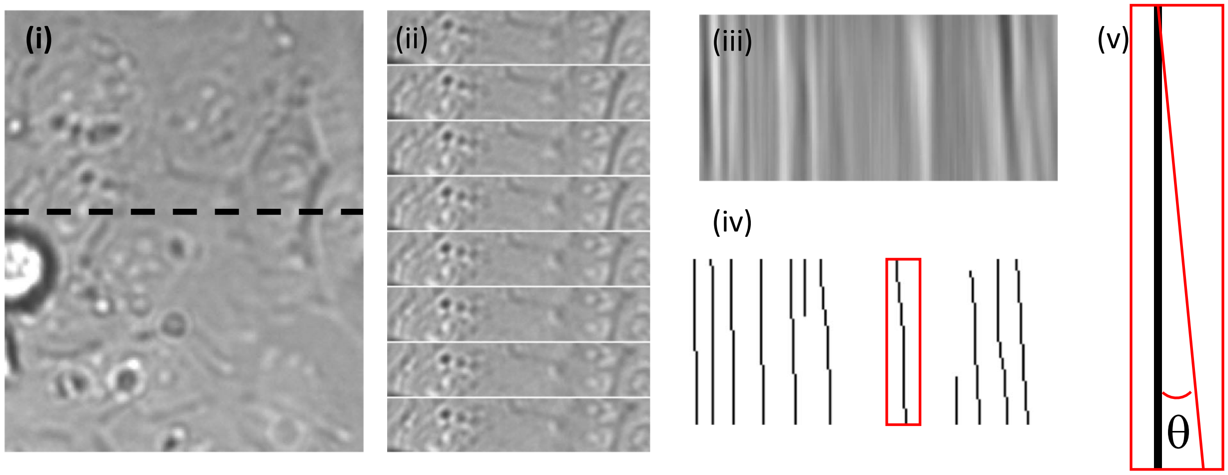

Supplement: Supplementary file 1 — Supplementary Information [file 41598_2019_40886_MOESM1_ESM.pdf]
